# Oral, June 9

**DOI:** 10.3402/ejpt.v4i0.21227

**Published:** 2013-06-04

## XIII ESTSS CONFERENCE: “Trauma and its clinical pathways: PTSD and beyond”, Bologna, June 2013 ORAL, JUNE 9 A/B PLENARY Hall

## Evidence-based practice on trauma
*
Symposium: SPE-STRESS - WHO guideline on stress related problems and disorders*

### Problems and disorders specifically related to stress (SPE-STRESS)—rationale and methods 8.45–9.05

#### M. Van Ommeren WHO, Geneva, Switzerland

The WHO's recently completed guideline “Problems and disorders specifically related to stress (SPE-STRESS)” was developed to address the absence of suitable, evidence-based guidelines for managing problems and disorders related to stress in primary health care and other non-specialised health care. This is the first presentation of this symposium on these guidelines and will cover the rationale and methods. There have been no suitable clinical guidelines for managing these mental health problems in primary health-care settings in low- and middle-income countries. Agencies working in post-conflict and natural disaster settings are increasingly interested in mental health care. This requires the development and testing of a module on the management of SPE-STRESS. The module would be part of the mhGAP programme, which is WHO's flagship programme for scaling up mental health care globally. An external Guideline Development Group (GDG) was formed to develop WHO recommendations based on systematic evidence appraisal of evidence (Barbui et al., 2010). WHO guideline development requires recent (not older than 2 years) systematic reviews of studies evaluating the interventions, with no date limitations on included individual studies. We searched for systematic reviews and if these were unavailable, commissioned them. The quality of evidence for each intervention was summarised using GRADE. Evidence profiles also listed judgments on: intervention benefits versus harms; values and preferences regarding the intervention (e.g., acceptability to end users); and feasibility of the intervention (e.g., resources needed). After peer review, the GDG met to agree on recommendations.

Reference

Barbui, C., Dua, T., van Ommeren, M., Yasamy, M. T., Fleischmann, A., Clark, N., et al. (2010). Challenges in developing evidence-based recommendations using the GRADE approach: The case of mental, neurological, and substance use disorders. *PLoS Med icine*.

### Problems and disorders specifically related to stress (SPE-STRESS)—pharmacological management 9.05–9.25

#### J. Bisson Cardiff University, Wales, UK

The WHO's recently completed guideline “Problems and Disorders Specifically Related to Stress (SPE-STRESS)” was developed to address the absence of suitable, evidence-based guidelines for managing problems and disorders related to stress in primary health-care and other non-specialised health-care settings. This is the second presentation of this symposium on these guidelines and will cover pharmacological management. Eight of SPE-STRESS's 21 recommendations concern pharmacological treatment. On the basis of the available evidence, the guideline recommends against the use of benzodiazepines in children, adolescents or adults to reduce acute traumatic stress symptoms or insomnia in the first month after a potentially traumatic event, or to assist with bereavement. Selective serotonin re-uptake inhibitors and tricyclic antidepressants are recommended to be considered in adults for the treatment of posttraumatic stress disorder (PTSD) if (1) stress management, cognitive-behavioural therapy with a trauma focus and/or eye movement desensitization and reprocessing have failed or are not available or (2) if there is concurrent moderate-severe depression. They are not recommended to manage PTSD in children and adolescents. Evidence from the systematic reviews and randomised controlled trials used to inform these recommendations will be presented along with the reasoning behind the recommendations and the strength of them.

### Problems and disorders specifically related to stress (SPE-STRESS)—psychological management 9.25–9.45

#### L. Jones Harvard School of Public Health, Boston, MA, USA

The WHO's recently completed guideline “Problems and disorders specifically related to stress (SPE-STRESS)” was developed to address the absence of suitable, evidence-based guidelines for managing problems and disorders related to stress in primary health-care and other non-specialised health-care settings. The third presentation will cover the13 of SPE-STRESS's 21 recommendations that concern psychological management. For adults these include recommendations forcognitive-behavioural therapy with a trauma focus to reduce acute traumatic stress symptoms in the first month after a potentially traumatic event. The guidelines recommend psycho-education and behavioral interventions for secondary non-organic enuresis and relaxation techniques and sleep hygiene for insomnia in the first month after exposure to traumatic events. No recommendations for psychological interventions could be made based on available randomised evidence with regard to dissociative symptoms and hyperventilation after a recent traumatic event. However, the guidelines warn against rebreathing in a paper bag for hyperventilation in children in the first month after exposure to a traumatic event. For adults and children with posttraumatic stress disorder (PTSD), recommended psychological treatments are cognitive behavioural therapy (CBT) with a trauma focus, eye movement desensitisation and reprocessing, and, in adults, stress management. The guidelines also recommend that structured psychological interventions should *not* be offered universally (to all) bereaved children and adults who do not meet the criteria for a mental disorder. The evidence used to inform these recommendations will be presented along with the reasoning behind them and their strength.

## Responding to disasters
*Symposium: Initiatives of the European Commission for target group oriented psychosocial aftercare programs - EUTOPA and EUNAD*

### Multidisciplinary guidelines on crisis intervention programs: what about disability management? 10:00–10:20

#### C. Schedlich^1^, G. Zurek^2^ and R. Bering^3^

^1^German Federal Office of Civil Protection and Disaster Assistance, Bonn, Germany; ^2^Alexianer-Institute for Psychotraumatology, Berlin Krefeld, Germany; ^3^Center of Psychotraumatology, Alexianer Krefeld GmbH, University of Cologne, Cologne, Germany

*Introduction*: In the last 10 years, the European Commission (EC) funded various projects, which aimed to develop and optimize quality standards and Multidisciplinary Guidelines (MGs) in psychosocial crisis management. However, most MGs focus early intervention. We are going to address the following questions: how are the different measures, interventions and resources linked to the needs of those affected? What are the actual approaches in solving interface problems for transition from acute to mid- and long-term psychosocial support? What has been done for minorities with special needs (e.g., handicapped people)? *Method*: A literature analysis has been conducted that is addressed to the questions if Pan-European projects have focused on the special needs of handicapped. *Conclusion*: Common terminology on measures and interventions of psychosocial crisis management is improving. However, the special needs of handicapped survivors of disasters have not frequently been taken into consideration.

### EUTOPA: European guideline for target group-oriented psychosocial aftercare-implementation: latest research on the validation of the target group intervention programme 10:20–10:40

#### R. Bering^1^, C. Schedlich^2^, D. Wagner^3^ and G. Zurek^3^

^1^Center of Psychotraumatology, Alexianer Krefeld GmbH/ University of Cologne, Cologne, Germany; ^2^German Federal Office of Civil Protection and Disaster Assistance, Bonn, Germany; ^3^Alexianer-Institute for Psychotraumatology, Krefeld Berlin, Germany

*Background*: The target group intervention programme (TGIP) is considered a secondary preventive concept of individual psychosocial aftercare and describes every intervention step from psychological primary care to indicated psychotherapy more specifically. Our concept is based on the opinion that process-orientation and identification of risk-groups is successful in driving forth effective crisis intervention programmes. However, the TGIP is not adapted to the special needs of handicapped. *Method*: The latest development on the TGIP is given compiled by demonstrating data from the brake down of the historical archive of Cologne as well as from the Love Parade Disaster in Duisburg. *Results*: Our field studies are in line with meta-analyses conducted for this purpose. Studies that address the questions of special risk factors of handicapped are rarely seen. *Conclusion*: Cumulative psychotraumatic exposure, peritraumatic dissociation, objective severity of the event, subjective evaluation of the event and reaction of the social and vocational environment are to be rated as ubiquitous factors which promote the development of stress disorders. However, special risk factors of target groups with special needs like blinds and deafs exist but they are not represented in risk factor models so far.

### EUNAD: assisting disabled in case of disaster 10:40–11:00

#### S. Vymetal^1^, A. Elklit^2^, T. Heir^3^, C. Schedlich^4^, K. Cummings^5^ and R. Bering^5^

^1^Charles University in Prague, Prague, Czech Republic; ^2^University of Southern Denmark, Odense, Denmark; ^3^Norwegian Centre for Violence and Traumatic Stress Studies, Oslo, Norway; ^4^German Federal Office of Civil Protection and Disaster Assistance, Bonn, Germany; ^5^Center for Psychotraumatology, Alexianer Krefeld GmbH, Germany

*Background*: The project EUNAD aims toward the implementation and preparation of EU human rights-related assistance programmes for disabled survivors of disasters. *Objectives*: The current project are: (1) evaluation of networks of associations for disabled; (2) conduction of qualitative studies on blinds and deafs in general psychotraumatology; (3) organisation of workshops to include associations for handicapped in the field of psychotraumatology; and (4) trainings of uniformed services, social workers and mental health professionals to assist handicapped after major incidents. Special attention is given to disaster management plans in israelian war zones. *Results*: EUNAD focus on recommendations for psychosocial support programs of deafs and blinds after disaster. *Conclusion*: EUNAD may be a step forward in the implementation of the UN Convention on the rights of persons with disabilities.

## Presidential Panel

### ESTSS Presidential panel: back to the future: trauma, Europe, and ESTSS 11.45–13.00

#### B. Gersons Academic Medical Centre, University of Amsterdam, The Netherlands

**Panel description**: What is the future of ESTSS in Europe and in the world? Past-presidents of ESTSS will answer in a lively debate questions posed by the Conference participants about trauma, Europe, and ESTSS. What does Europe mean for the former ESTSS presidents? Is ESTSS for research or also for debate and clinical practice? Putting participants’ questions will be two excellent young ESTSS members Evaldas Kazlauskas (Lithuania) and Mirjam Nijdam (The Netherlands). The participating presidents are: Stuart Turner (UK), Roderick Orner (UK), Ueli Schnyder (Switzerland), Dean Ajdukovic (Croatia), Berthold Gersons (The Netherlands), Jonathan Bisson (UK), Miranda Olff (The Netherlands), Brigitte Lueger-Schuster (Austria), and Vedat Sar (Turkey).

**Panelists**: ESTSS Past Presidents and current President

## ORAL, JUNE 9 HALL DIAMANTE

## Invited Symposium: Trauma-related psychopathology and service delivery in forensic mental health services

### Prevalence on complex PTSD in prison settings and a strategy for trauma-informed services 8:45–9:05

#### V. Ardino^1^ and L. Milani^2^

^1^PSSRU Unit, London School of Economics and Political Science, London, UK; ^2^CRidee, Dipartimento di Psicologica, Universitá Cattolica del Sacro Cuore, Milano, Italy

This paper investigates the main psychological and criminological issues underlying the definition, measurement, and treatment of trauma and post-traumatic reactions in forensic settings with relevant reference to research and to possible pathways of care in forensic settings. The paper will derive from current prevalence data on complex PTSD a few insights into the implementation of innovative management strategies to include the notion of trauma-informed services within forensic services. The evidence demonstrated that prisoner populations present a wide spectrum of childhood interpersonal trauma; therefore, specific aspects of early trauma as measured highlight different pathways to CPTSD and re-offending risk that should be considered in rehabilitation programs.

### Trauma and psychopathy 9:05–9:25

#### V. Caretti^1^, A. Schimmenti^2^, G. Craparo^2^ and G. Di Carlo^1^

^1^Department of Psychology, University of Palermo, Palermo, Italy; ^2^Kore University, Enna, Italy

*Objectives*: The relationship between traumatic experiences and antisocial personality disorder is well estabilished; the same cannot be said about the relationship between traumatic experiences and psychopathy (Hare, 1999). In fact, although several authors have suggested that the origin of psychopathic personality can be rooted in adverse relational experiences with caregivers during childhood (e.g. McWilliams, 2011), research on this issue appears to be inconclusive. The study analyzes the relationship between traumatic experiences and psychopathy in a subset of the Italian PCL-R validation sample (Caretti, Manzi, Schimmenti, & Seragusa, 2011) who answer questions on traumatic experiences; case studies will be also presented to elucidate such relationship. *Method*: The sample involved 121 Italian offenders (85% males) who were convicted for violent crimes. The sample was recruited in prisons and forensic psychiatic facilities. Age in this sample ranged from 23 to 71 (M=42, SD=10). Two measures were administered to the sample: (1) Psychopathy Checklist-Revised (PCL-R; Hare, 2003). The PCL-R is a 20-item clinician-report measure to assess psychopathy and its related psychological and behavioral aspects. (2) Traumatic Experiences Checklist (TEC, Nijenhuis et al., 2002). The TEC is a 29-item self-report measure used to assess a wide range of potential traumatic experiences from childhood to adulthood. *Results*: Partial correlations were used to analyze the associations between PCL-R scores and the TEC total scores, controlling for age. Traumatic experiences significantly correlated with PCL-R total scores (*r*=0.34, *p*<0.001), Factor 1 scores (*r*=0.18, *p*<0.05) and Factor 2 scores (*r*=0.41, *p*<0.001). Regression analyses showed indeed that TEC scores were able to predict the PCL-R Total score (*F*(1,19)=15.70, *p*<0.0001, R^2^=0.12). *Discussion*: Findings of the study show that traumatic experiences play a key role in the development of personality disorders; however, the stronger associations were found between traumatic experiences and the social deviance factor of the PCL-R (Factor 2). It is then possible that other variables (including genetic and temperamental ones) have a mediating role in linking traumatic experiences and the interpersonal and affective facets of psychopathy. Thus, case studies from the PCL-R interviews will be presented in order to illustrate some of the possible developmental pathways from traumatic experiences to psychopathy.

References

Caretti, V., Manzi, G. S., Schimmenti, A., & Seragusa, L. (2011). PCL-R. Hare Psychopathy Checklist-Revised. Firenze, Italy: OS Giunti.

Hare, R. D. (1999). Without conscience: The disturbing world of the psychopaths among us. New York, NY: Guilford Press.

Hare, R. D. (2003). Hare's Psychopathy Checklist Revised (PCL-R)—Second Edition. Technical Manual. Toronto, ON, Canada: Multi-Health Systems.

McWilliams, N. (2011). Psychoanalytic diagnosis, second edition: understanding personality structure in the clinical process. New York, NY: Guilford Press.

### Mental health services in-reach and trauma 9:25–9:45

#### A. Forrester^1,2^

^1^South London & Maudsley NHS Foundation Trust, London, UK; ^2^Department of Forensic & Neurodevelopmental Science, Institute of Psychiatry, King's College London, London, UK

In England and Wales, mental health in-reach services have been developed and delivered into prisons over the last 10 years or more. This delivery followed the transfer of delivery arrangements from the Home Office to the National Health Service (NHS), which provides the majority of healthcare within the United Kingdom. This new form of delivery has been driven by policy, in keeping with the principle of equivalence (Exworthy et al., 2012) and as a consequence many new services are now in place. More recently, since an influential national report by Lord Bradley (Department of Health, 2009), there has been enhanced emphasis on services working across criminal justice pathways, including court liaison and diversion services and newer services working in police custody areas. The background to these policy initiatives is presented (Home Office, 1996; Home Office & NHS Working Group, 1999; Home Office, 2007; Department of Health, 2009) along with a description of the development of services over the last decade or more (e.g., Steel et al., 2007). Recent evaluations of local prison in-reach teams are presented (Forrester et al., 2010, Forrester et al., in submission), along with the results of a new national survey of prison mental health services (Forrester et al., in press). Ensuring that services follow policy recommendations has led to linkage between different areas within offender mental health, often with the same individuals working across a range of criminal justice areas. This has led to service developments working across police stations, courts, prisons, and probation, in order to ensure that the healthcare pathways inside justice systems are as inter-connected as possible. Local examples of this type of service development are described, along with unpublished results from a first cohort of over 500 people to receive mental health services while detained in police custody settings in an area of South London. Research in this area is limited, but early results support the view that there are high levels of mental health morbidity and health service disengagement among this group.

References

Department of Health. (2009). Lord Bradley's review of people with mental health problems or learning disabilities in the criminal justice system. Department of Health: London.

Exworthy, T., Samele, C., Urquia, N., & Forrester, A. (2012). Asserting prisoners’ right to health: progressing beyond equivalence. *Psychiatric Services*, *63*, 3.

Forrester, A., Chiu, K., Dove, S., & Parrott, J. (2010) Prison health-care wings: Psychiatry's forgotten frontier. *Criminal Behaviour and Mental Health*, *20*, 5161.

Forrester, A., Exworthy, T., Olumoroti, O., Sessay, M., Parrott, J., Spencer, S. J., et al. (in press). Variations in prison mental health services in England and Wales. *The International Journal of Law and Psychiatry*.

Forrester, A., Singh, J., Ardino, V., Slade, K., Samele, C., Exworthy, T., et al. (in submission). Prison mental health in-reach services after more than a decade of development in England. *European Journal of Psychotraumatology*.

HM Prison Service & NHS Executive Working Group (1999). The Future Organisation of Prison Health Care. London: Department of Health.

Home Office. (1996). Patient or Prisoner? Home Office: London.

Home Office. (2007). A Report by Baroness Jean Corston of a Review of Women with particular vulnerabilities in the Criminal Justice System. Home Office: London.

Steel, J., Thornicroft, G., Birmingham, L., Brooker, C., Mills, A., Harty, M., et al. Prison mental health inreach services. *British Journal of Psychiatry*, *190*, 373–374.

## Open Papers: Family processes

### Ambiguous loss, PTSD and marital relations 10:00–10:15

#### R. Dekel Bar Ilan University, Ramat Gan, Israel

*Background*: There is a solid base of evidence that posttraumatic symptoms (PTS) following military conflicts are associated with lower marital satisfaction and higher distress among female spouses. Earlier qualitative studies suggested the concepts of ambiguous loss and boundary ambiguity to describe the situations in which the veteran returns home from deployment and copes with PTS. The current quantitative study examines the role of boundary ambiguity as a mediator between veterans’ PTS and spouses’ adjustment. *Methods*: Two hundred and forty couples took part in the current study. In all cases, the male participated in combat. While half of them were referred to mental health services after returning home, the other half were not. Veterans completed a posttraumatic stress disorder (PTSD) questionnaire. Spouses completed the following questionnaires: Boundary ambiguity, well-being, functioning and secondary traumatization. Personal and couples’ background data were assessed. *Results*: Boundary ambiguity was a full mediator between veterans’ PTS and female spouses’ well-being and functioning and a partial mediator between veterans’ PTS and spouses’ secondary traumatization symptoms. *Discussion*: The results highlight the applicability of ambiguous loss as a useful model for understanding and treating families in which one of the spouses suffers from PTS. In addition, it clarifies several pathways through which PTS affects various dimensions of spouses’ adjustment.

### Posttraumatic outcome of intimate partner violence: aggregate effects of childhood maltreatment and cognitive distortions 10:15–10:30

#### J. Henrie, P. Petretic, M. Karlsson and M. Calvert Department of Psychology, University of Arkansas, Fayetteville, AR, USA

Studies have revealed that approximately 58–64% of female victims of intimate partner violence (IPV) experienced posttraumatic stress disorder (PTSD) symptoms (e.g., Astin et al., 2002). Given the variability of IPV posttraumatic outcome, it is incumbent to identify the mechanisms through which PTSD symptomatology develops. Extant research hypothesizes that multiple childhood trauma experiences (e.g., various forms of maltreatment) have additive effects on later trauma (e.g., Cloitre et al., 2009). Furthermore, a strong association between maladaptive cognitions and PTSD symptoms has been identified (e.g., Belsher et al., 2012). This study examines the aggregate risk effects of prior maltreatment experiences and maladaptive cognitive styles on subsequent PTSD symptomatology following IPV victimization. In the authors’ existing sample of college students (*N*=762), hierarchical multiple regression analyses revealed a significant model [*F*(9, 400)=67.387, *p*<0.001. Adjusted *R* ^2^=0.594];their separate sample of female college students (*N*=189) also revealed a significant model [*F*(10, 128)=19.47, *p*<0.001. Adjusted *R* ^2^=0.572]. In both samples, each level of predictor variables was significant. Currently, there are no empirical studies testing this model within a community sample of adult females, despite research indicating a broader range of negative impact identified within adult community samples versus college convenience samples. Thus, the current project will test this model in a large community sample of adult females. In addition to a demographic forms, the respondents will complete the following measures: Revised Conflict Tactics Scale (IPV exposure), Trauma Symptom Inventory-2 (PTSD symptomatology), Childhood Maltreatment Interview Schedule Short Form (childhood maltreatment), and Cognitive Distortion Scales (maladaptive cognitions). Data collection is underway. Findings will be compared and contrasted with those of the aforementioned college samples, such that the applicability of the larger theoretical model can be discussed across demographically diverse samples.

### Co-brooding in the couple: repetitive negative sharing as a risk factor for adjustment disorder 10:30–10:45

#### A. Horn and A. Maercker Division of Psychopathology and Clinical Intervention, University of Zurich, Zurich, Switzerland

In the individual, brooding has been identified as the maladaptive component of rumination predicting adverse mental health outcomes. As soon as the brooding-related thoughts and feelings are shared repetitively – e.g. with the romantic partner – it can be seen as co-rumination. The aim of this study was to study the predictive value of co-rumination in the couple as a risk factor for symptoms of adjustment disorder after an adverse life event above and beyond established individual risk factors. Of 334 individuals who participated in an online-couple study, *N*=174 reported having experienced an adverse event. Co-brooding in the couple was assessed with a new questionnaire, beside intrapersonal brooding and emotion regulation strategies. Adjustment disorder symptoms were assessed with the ADNM concurrently, and 3 months later. Results reveal that co-brooding predicted adjustment disorder symptoms longitudinally above and beyond known intrapersonal risk factors. The results underline the importance of socio-interpersonal processes as risk and protective factors in the aftermath of an adverse event that might be worth exploring further.

### Childbirth, PTSD and past traumatic experience: a complex relationship 10:45–11:00

#### S. Freedman^1^, R. Casif-Lerner^2^, U. Elhalal^3^ and C. Weiniger^4^

^1^Bar Ilan University, Ramat Gan, Israel; ^2^Medical School, Hadassah-Hebrew University Medical Center, Ein Kerem, Jerusalem, Israel; ^3^Department of Obstetrics and Gynecology, Hadassah-Hebrew University Medical Center, Ein Kerem, Jerusalem, Israel; ^4^Department of Anesthesiology and Critical Care Medicine, Hadassah-Hebrew University Medical Center, Ein Kerem, Jerusalem, Israel

*Background*: posttraumatic stress disorder (PTSD) following childbirth occurs in approximately 2% of the cases, and is associated with previous experiences of child sexual abuse (CSA) and traumatic events during birth itself. The impact of other previously experienced traumatic events and antenatal behavior on PTSD has not been examined. *Method*: This self-report cross-sectional study examined 185 women. Participants were 12–48-hour postpartum women in the postnatal wards. A trained investigator presented the questionnaires and the anonymous completion strategy, with questionnaires returned to a locked box. Questionnaires assessed trauma history, peritraumatic dissociation, PTSD and depression symptoms, and a birth questionnaire. *Results*: CSA was not related to elevated symptom levels. Birth-related trauma was related to significantly higher depression and dissociation scores, although not with elevated PTSD levels. Women reporting previous (but not current) traumatic birth, were significantly less likely to have taken a doula (3.3%, past birth traumatic vs. 31% no birth traumatic, χ^2^=12.7, *p*<0.05) and were significantly more likely to have requested epidural analgesia (75.93% past birth traumatic vs. 57.5% no birth traumatic, χ^2^=7.6, *p*<0.05). *Conclusions*: These results may indicate that previous experience of traumatic events other than CSA, particularly regarding birth experience, may affect postpartum reactions, and that birth-related trauma may affect decisions regarding consequent birth plans and posttraumatic reactions.

## ORAL, JUNE 9 HALL FALCO

## The spectrum of trauma-related disorders
*
Symposium: Intrusive re-experiencing - New developments in experimental and clinical approaches*

### Capturing intrusive re-experiencing in trauma survivors’ daily lives using ecological momentary assessment 8:45–9:00

#### B. Kleim^1^, B. Graham^2^, R. Bryant^3^ and A. Ehlers^4^

^1^University of Zurich, Zurich, Switzerland; ^2^University College London, London, UK; ^3^University of New South Wales, Sydney, Australia; ^4^University of Oxford, Oxford, UK

Intrusive memories are common following traumatic events and among the hallmark symptoms of posttraumatic stress disorder (PTSD). Most studies assess summarized accounts of intrusions retrospectively. We used an ecological momentary approach and index intrusions in trauma survivors with and without PTSD using electronic diaries. Forty-six trauma survivors completed daily diaries for seven consecutive days recording a total of 294 intrusions. Participants with PTSD experienced only marginally more intrusions than those without PTSD, but experienced them with more “here and now quality,” and responded with more helplessness and shame than those without PTSD. Most frequent intrusion triggers were stimuli that were perceptually similar to stimuli from the trauma. Individuals with PTSD experienced diary-prompted voluntary trauma memories with the same sense of nowness and vividness as involuntary intrusive trauma memories. The findings contribute to a better understanding of everyday experiences of intrusive re-experiencing in trauma survivors with PTSD and offer clinical treatment implications.

### Memory reactivation and the onset of intrusive memories 9:00–9:15

#### R. Bryant and J. Cheung School of Psychology, University of New South Wales, Sydney, Australia

It is well established that stress hormones affect the consolidation and retrieval of emotional memories. Recent evidence also suggests a role for stress hormones in the reconsolidation of emotional memories, which holds promise for the treatment of disorders such as posttraumatic stress disorder (PTSD). The extent to which the processes underpinning memory reactivation impact on intrusive memories has yet to be explored. The current study examined the effect of endogenous stress hormones on intrusive memories of a previously encoded emotional event. Sixty-three healthy participants viewed a highly stressful film, and two days later were exposed to (1) a cold water stressor prior to reactivation of the encoded memory, (2) a non-stress condition prior to memory reactivation, or (3) a cold water stressor without memory reactivation. Reactivation following a stressor led to greater intrusions of the distressing film. These findings suggest that intrusive memories of a trauma that may be compounded by stressors that occur in the aftermath of the trauma and in the context of remembering the traumatic event. Implications for managing traumatic intrusions are discussed.

### The effects of mental imagery on intrusion development and automatic defense responses 9:15–9:30

#### M. A. Hagenaars^1^, H. Cremers^1^ and A. Arntz^2^

^1^Radboud University Nijmegen, Nijmegen, The Netherlands; ^2^Maastricht University, Maastricht, The Netherlands

The importance of mental imagery in clinical psychology has received a lot of attention in the past decade, underscored by the appearance of several special issues on this topic. Several theories have suggested a special role for mental imagery versus abstract contextualized memories with associated distinct neurobiological structures being involved in both processes (Brewin, Gregory, Lipton, & Burgess, 2010; Holmes & Mathews, 2010). As a feature of emotional memory in general, mental imagery is considered to play an important role in the development and maintenance of a wide range of psychiatric disorders. Experimental studies help to unravel the underlying mechanisms. Here, two experiments are presented that investigated the effects of posttrauma imagery rescripting on the development of intrusive memories using a trauma analog design. In the first study, the effects of three interventions (imagery re-experiencing, imagery rescripting, and positive imagery, conducted 30 minutes posttrauma) were examined, resulting in clear differences in intrusion development. In the second study, these results were replicated for the two major interventions and the role of individual differences (pretrauma anxiety symptoms) was taken into account. Furthermore, a third experiment showed that imagery rescripting also influences automatic defense responses such as the freezing response, indicating its importance as a tool in the treatment of psychopathology.

References

Brewin, C. R., Gregory, J. D., Lipton, M., & Burgess, N. (2010). Intrusive images in psychological disorders: characteristics, neural mechanisms, and treatment implications. *Psychological Review, 117*(1), 210–232.

Holmes, E. A., & Mathews, A. (2010). Mental imagery in emotion and emotional disorders. *Clinical Psychology Review, 30*(3), 349–362.

### Intrusive memories of hallucinations and delusions after intensive care 9:30–9:45

#### D. Wade^1^, C. Brewin^2^ and J. Weinman^3^

^1^Critical Care, University College Hospital, London, UK; ^2^Department of Psychology, University College London, London, UK; ^3^Department of Psychology, Kings College London, London, UK

*Background*: Many patients have frightening hallucinations in intensive care units (ICUs), and there is a high prevalence of posttraumatic stress disorder (PTSD) among ICU survivors.^1^ Previous studies found that patients experienced “delusional” or “factual” memories after leaving the ICU.^2^ We aimed to investigate the prevalence, nature and content of ICU-related intrusive memories as this has not previously been done. *Methods*: The prevalence of early intrusive memories and amnesia in intensive care were measured in a prospective cohort study of 157 patients,^3^ among other psychological and clinical risk factors for PTSD. A sample of patients who indicated intrusive memories on the Posttraumatic Diagnostic Scale (PDS) three months after ICU discharge were later interviewed using the Full Intrusions Interview (*n*=17). *Results*: It was found that 65% of the cohort had hallucinations, and 47% had early intrusive memories of the ICU before discharge. More than half of early intrusive memories included hallucinations experienced in ICU. Furthermore, the 45% of patients who remembered little of their ICU stay were more likely to have early intrusive memories (62% vs 39%, *p*=0.02). Associations were also found between receiving benzodiazepines in the ICU and delirium, hallucinations, amnesia and intrusive memories. At three months post-ICU, 27% of patients had scores over 18 on the PDS, indicating PTSD. When content analysis of the 17 interviews was carried out, 15 patients’ intrusive memories were of hallucinations and paranoid delusions from intensive care; while two patients’ memories were mainly factual, such as bleeding or pain. The themes of hallucinatory intrusions included torture, courtrooms, cults, gas-chambers, zombies; and conspiracies by hospital staff to steal patients’ organs, blood, money, or souls. Memories were rated as vivid, clear, frequent, long-lasting, uncontrollable and distressing, with helplessness and anxiety. *Conclusion*: Many patients re-experience frightening hallucinations and delusions as intrusive memories months after leaving an ICU.

## Psychobiology and PTSD
*
Symposium: The roles of neuroendocrine stress responses in the process of traumatic memory*

### Glucocorticoid signaling, traumatic memories and posttraumatic stress symptoms in survivors of critical illness and intensive care therapy 10:00–10:20

#### D. Hauer and G. Schelling Department of Anaesthesia, University of Munich, Munich, Germany

Critically ill patients are at an increased risk for traumatic memories and posttraumatic stress disorder (PTSD) and often receive exogenously administered glucocorticoids for medical reasons. Critical illness could therefore represent a useful model for investigating HPA-axis functioning and glucocorticoid effects on traumatic memories and PTSD development. Studies in long-term survivors of intensive care units (ICU) treatment demonstrated a clear and vivid recall of traumatic experiences and the incidence and intensity of PTSD symptoms increased with the number of traumatic memories present. Recent experiments in animals have clearly shown that the consolidation and retrieval of traumatic memories is regulated by an interaction between the glucocorticoid and the endocannabinoid system (ECS). The ECS is also an important regulator of the HPA-axis’ activity during stress *per se*, an effect which has also been demonstrated in humans. Likewise, a single nucleotide polymorphisms (SNP) of the glucocorticoid receptor (GR) gene (the Bc*l*I–SNP) which enhances the sensitivity of the GR to cortisol and possibly HPA-axis feedback function was associated with enhanced emotional memory performance in healthy volunteers. The presence of the Bc*l*I–SNP also increased the risk for traumatic memories and PTSD symptoms in patients after ICU therapy and was linked to lower basal cortisol levels. Interestingly, the prolonged administration of glucocorticoids to critically ill patients resulted in a significant reduction of PTSD symptoms measured after recovery without influencing the number of categories of traumatic memory. This effect was also seen after a single bolus of hydrocortisone in individuals after exposure to a highly traumatic event. These hydrocortisone effects can possibly be explained by a cortisol-induced temporary impairment in traumatic memory retrieval which has previously been demonstrated in both rats and humans. Stress doses of hydrocortisone or the pharmacologic manipulation of glucocorticoid–endocannabinoid interaction during traumatic memory consolidation and retrieval could be useful for prophylaxis and treatment of PTSD.

### Examining the moderating roles of the cardiac defense response and salivary alpha-amylase in the relationship between cortisol and traumatic intrusions 10:20–10:40

#### C. Chou^1^, R. La Marca^2^, A. Steptoe^3^ and C. Brewin^1^

^1^Clinical Psychology, University College London, London, UK; ^2^Klinische Psychologie und Psychotherapie, Universität Zürich, Zurich, Switzerland; ^3^Institute of Epidemiology and Health Care, University College London, London, UK

*Objectives*: Cortisol has been extensively studied in relation to posttraumatic stress disorder (PTSD). Inconsistent findings have suggested the need to investigate possible moderators. This study assessed cortisol levels during memory encoding and consolidation with the Trauma Film Paradigm. The relationship of cortisol with intrusive memory was assessed. Cardiac defense response (CDR) and salivary alpha-amylase (sAA), indicators of stress coping patterns and sympathetic activation, respectively, were examined as moderators. *Methods*: The CDR was assessed to a group of 45 healthy adult participants as Accelerators (who are more physiologically prepared to confront or escape from stressors) or Decelerators. Pre-existing psychological characteristics were assessed in all participants before they were introduced to the trauma film. Saliva samples were collected at baseline, and at the encoding and consolidation stages of the film. The frequency and vividness of intrusive memories for the film were recorded with an intrusion diary for seven days. *Results*: Cortisol increased in response to the film. Participants with higher pre-existing posttraumatic distress released lower level of cortisol post-film. In terms of the association with intrusive memory, lower cortisol at the encoding and consolidation stages predicted greater vividness of intrusions. The correlations of cortisol and intrusion frequency were moderated by the CDR at the encoding stage, and sAA levels at the early consolidation stage. *Conclusions*: Inconsistency between the findings concerning intrusion vividness and frequency imply diverse mechanisms underlying the two measures. The associations between cortisol, posttraumatic distress and intrusion vividness support literature arguing an insufficiency of cortisol as a cause of over-consolidation of traumatic memory (Yehuda & Harvey, 1997). Moreover, the moderating effects suggested the importance of considering individual differences in stress-coping patterns and sympathetic activation when studying the role of cortisol in traumatic memory.

### The role of frontal brain asymmetry in physiological responding to negative memories 10:40–11:00

#### T. Meyer, C. Quaedflieg and T. Smeets Department of Clinical Psychological Science, Maastricht University, Maastricht, The Netherlands

Frontal brain asymmetry is an electroencephalography (EEG) biomarker that can serve as an indicator of psychopathology, as well as of adaptive responding to adverse experiences. For instance, relatively left-sided frontal activity has been shown to predict better self-regulation of startle responses to negative pictures. We investigated whether frontal asymmetry would dampen startle responses during and after aversive memory elicitation. Resting EEG was recorded in 64 participants, followed by presentation of one of two trauma films with continuous EEG recordings. Afterwards, participants underwent an auditory startle paradigm including neutral pictures of both trauma films (one of which was previously seen) and unrelated neutral pictures. We expected more left-sided frontal activity at rest and during film viewing to correlate with dampened startle reactions in response to film-related memory elicitation. We will present our preliminary findings and discuss potential implications for the prediction of posttraumatic stress disorder (PTSD) after aversive experiences.

## ORAL, JUNE 9 HALL GARGANELLI

## Open Papers: Impact of trauma on communities

### Utilization of societal trauma in post-conflict societies: contemporary version of “Enemy of the People” image in the South Caucasus 8:45–9:00

#### J. D. Javakhishvili^1,2^

^1^Ilia State University, Tbilisi, Georgia; ^2^Foundation Global Initiative on Psychiatry, Tbilisi, Georgia

This paper presents meta-analyses of the research revealing patterns of utilization of societal trauma and associated with it phenomena in internal socio-political life in the six post-conflict societies of the South Caucasus (Georgian, Armenian, Azerbaijani, Abkhaz, Nagorno-Karabakhian and South Ossetian). The research took place in 2012, in the frame of the International Alert's “Mediation & Dialogue in South Caucasus” Program, funded by EU. A multi-ethnic team of researchers, representing each country/constituency of the South Caucasus was involved in the study. Qualitative methods such as discourse analysis, in-depth interviews and case studies were used. The meta-analyses reveals the presence of societal trauma and “Enemy Image” phenomenon in current socio-political discourses of the South Caucasus societies; it shows how the trauma is transmitted to the generation, which has not witnessed the armed conflicts/wars via different types of narratives; it shows how maladaptive cognition shared within the society on the necessity/usefulness of enemy image transforms it into powerful manipulation tool, utilized by the governing elites and other political forces active in the countries/societies; it describes how the “Enemy of People” concept inherited from the Soviet system obtained a new frame and is projected onto political opponents, bridging current socio-political life of the societies with the Soviet past. The comparative analysis of the patterns of utilization of the societal trauma-related phenomena in the six societies and their impact on internal socio-political life will be presented, an experience of particularly designed trauma-recovery focused reconciliation activities in South Caucasus and corresponding lessons learned out of them will be shared.

Reference

Javakhishvili, J. D., Kvarchelia, L. (editors), Abasov, I., Bagdasaryan, G., Hovanesyan, M., Kabulov, E., Khapava, K. (contributors). 2013. *Myths and conflicts: Instrmentalisation of conflict in political discourse*. London, UK: International Alert.

### The problem of continuous trauma in dislocated post-apartheid communities: preliminary findings of community projects based on COR theory 9:00–9:15

#### G. Van Wyk Traumaclinic Emergency Counselling Network, Cape Town, South Africa

Many communities that were dislocated under apartheid in South Africa are still trapped in spirals of poverty, violence, abuse, and neglect, with high levels of continuing trauma exposure. Current and best practice treatment methods are not accessible, affordable, or efficient in dealing with traumatic stress on this scale. The typical Western, individualised approach, dealing only with past trauma, is also not appropriate in these communities. In this paper, it is proposed that the Conservation of Resources Theory (COR), with its accent on social support resources, may provide a model for designing pragmatic interventions in these communities and possibly also in other under-resourced communities. The preliminary findings of a number of such community projects in Cape Town are discussed.

### Readiness to reconcile and mental health in internally displaced persons in Colombia 9:15–9:30

#### N. Stammel^1^, C. Heeke^2^, M. Ziegler^3^, M. T. Diaz Gomez^1^ and C. Knaevelsrud^1^

^1^Center for Torture Victims, Free University of Berlin, Berlin, Germany; ^2^Center for Torture Victims, Berlin, Germany; ^3^Center for Torture Victims, Humboldt University Berlin, Berlin, Germany

*Background*: The armed conflict in Colombia has led to severe human rights violations, forced displacements and other forms of violence in about one-third of the population since 1960. In recent years, the Colombian government adopted different reparation measures to indemnify the victims of the conflict and to provide peace and reconciliation in the country. Worldwide, reconciliation has become a key concept for sustainable peace activities in post-conflict societies. Recent studies provide evidence for a positive relationship between readiness to reconcile and mental health among victims of human rights violations. *Method*: *N*=454 randomly selected internally displaced persons were interviewed in a cross-sectional study in four provinces of Colombia. We assessed symptoms of posttraumatic stress disorder (PTSD), anxiety and depression as well as the participants’ readiness to reconcile with the perceived perpetrators in structured interviews. *Results*: Preliminary analysis did not show significant relationships between readiness to reconcile and sociodemographic or displacement-specific variables. There were, however, significant negative relationships between readiness to reconcile and symptoms of PTSD (*r*=−0.17, *p*<0.01), depression (*r=*−0.15, *r*<0.01) and anxiety, respectively. *Discussion*: The preliminary results are in line with international research on the relationship between readiness to reconcile and mental health. The results will be discussed in the context of international studies and the current political situation in Colombia.

### Psychiatric diagnosis and traumatic experiences among men seeking treatment for violent behaviour against their partner 9:30–09:45

#### I. R. Askeland, B. Loemo, J. Strandmoen, O. A. Tjersland and T. Heir Norwegian Centre for Violence and Traumatic Stress Studies, NKVTS, Oslo, Norway

*Objectives*: Traditionally, treatment programs for intimate partner violence (IPV) have been modeled as a “one size fits all.” The aim of this presentation is to present data on the prevalence of psychiatric diagnosis and potentially traumatic experiences among men using IPV and its implications for treatment. *Methods*: The Traumatic Experiences Checklist and the Mini International Neuropsychiatric Interview (MINI 6.0.0) were administered in a pretreatment clinical interview of 192 men who voluntarily attended treatment for IPV. *Results*: The majority of the men (70.1%) fulfilled the diagnostic criteria for at least one psychiatric diagnosis, measured by MINI. Nearly 2 out of 10 (18.5%) qualified for a PTSD diagnosis. The majority of the men (76.2%) reported potential traumatic experiences in their family of origin. Half of the men had experienced emotional neglect (49.2%). Six out of 10 (61.8%) had experienced physical abuse from their parents or older siblings. *Discussion*: Associations between diagnoses and reported traumatic experiences will be discussed. The high prevalence of psychiatric diagnosis in this group might indicate a need to screen for psychiatric symptoms and to implement an individually tailored treatment. Clinical pathways will be illustrated by presenting a single case.

### A collaborative model for building capacity in post-conflict mental health services 9:45–10:00

#### E. Newnham^1^, A. Akinsulure-Smith^2^, K. Hann^1^, N. Hansen^3^ and T. Betancourt^1^

^1^Harvard School of Public Health, Boston, MA, USA; ^2^City University of New York, New York, NY, USA; ^3^Yale University, New Haven, CT, USA

Mental health disorders contribute to a vast proportion of the global burden of disease, yet this need is largely unmet in many post-conflict settings. A critical element of bridging this gap entails addressing human resources constraints and ensuring high-quality training and supervision of local mental health workers. The presentation will describe training practices for an evidence-based, group mental health intervention for war-affected youth in Sierra Leone. Clinical training was conducted over a two-week period. Sessions comprised didactic learning, intensive role play, and within group-feedback on intervention components, including psychoeducation for trauma, sequential problem solving, interpersonal and communication skills, behavioral activation, and cognitive restructuring. A collaborative approach to training and implementation was vital: for each technique, trainees contributed locally relevant examples and context which informed delivery of the treatment. Supervision was conducted in-country and via weekly Skype or phone meetings with an international team. Analysis of the trainees’ fidelity to the intervention and supervision records illustrated the strengths of a collaborative training model. A strong grounding in evidence-based practice, and guided culturally relevant implementation of the intervention, highlighted a model applicable to other limited-resource settings.

## Open Papers: Military research

### What explains Posttraumatic Stress Disorders (PTSD) in UK service personnel: deployment or something else? 10:00–10:15

#### M. Jones^1^, J. Sundin^2^, L. Goodwin^1^, L. Hull^1^, N. T. Fear^1^, S. Wessely^1^ and R. J. Rona^1^

^1^King's Centre for Military Health Research, King's College, London, UK; ^2^Academic Centre for Defence Mental Health, King's College, London, UK

Unlike US studies, UK studies have not found an overall “deployment effect” on the prevalence of PTSD in regular armed forces personnel deployed to Iraq or Afghanistan. The aims of the current study were to assess whether the lack of difference in PTSD prevalence between the group deployed to Iraq or Afghanistan and the comparison group can be explained by the inclusion, in the comparison group, of personnel who have deployed elsewhere and who have a high rate of PTSD; and to assess the factors associated with PTSD in those not deployed, deployed to Iraq and/or Afghanistan or deployed elsewhere. The sample comprised 8261 regular UK armed forces personnel who deployed to Iraq, Afghanistan, other operational areas or were not deployed. Deployment to Iraq or Afghanistan (OR 1.2, 95% CI 0.6–2.2) or elsewhere (OR 1.1, 0.6–2.0) was unrelated to PTSD although holding a combat role was associated with PTSD if deployed to Iraq or Afghanistan (OR 2.7, 1.9–3.9). Childhood adversity (OR 3.3, 2.1–5.0), having left service (OR 2.7, 1.9–4.0) and serious accident (OR 2.1, 1.4–3.0), were associated with PTSD while higher rank was protective (OR 0.3, 0.12–0.76). For the majority of UK armed forces personnel, deployment confers no greater risk for PTSD than service in the armed forces per se. Vulnerability factors such as lower rank, childhood adversity and leaving service, and having had a serious accident may be at least equally important as holding a combat role in predicting PTSD in UK armed forces personnel.

### Predicting persistent PTSD in the UK military who were deployed to Iraq: a longitudinal study 10:15–10:30

#### R. Rona^1^, M. Jones^1^, J. Sundin^2^, L. Goodwin^1^, L. Hull^1^, S. Wessely^1^ and N. Fear^2^

^1^Kings College London, Kings Centre for Military Health Research, London, UK; ^2^Kings College London, Academic Centre for Defence Mental Health, London, UK

The purpose of this study was to assess whether it was possible to distinguish between short lived and persistent posttraumatic stress disorder (PTSD) over a mean period of three years. We assessed which baseline risk factors are associated with persistent and partially remitted PTSD in comparison to fully remitted PTSD. A randomly selected sample of 6427 (68%) UK service personnel completed the PTSD checklist (PCL) between 2004 and 2006 (Phase 1) and between 2007 and 2009 (Phase 2). Two hundred and thirty (3.9%) had possible PTSD at baseline. 66% of those with possible PTSD at baseline remitted (PCL score<30) or partially remitted (PCL score 30–49) by Phase 2 of the study. Associations of persistent PTSD, compared to the fully remitted group, with risk factors at Phase 1 adjusted for confounders were having discharged from service (OR 2.97, 95% CI 1.26–6.99); higher educational qualification (OR 2.74, 95% CI 1.23–6.08); feeling unsupported on return from deployment (OR 10.97, 95% CI 3.13–38.45); deployed but not with parent unit (OR 5.63, 95% CI 1.45–21.85); multiple physical symptoms (OR 3.36, 95% CI 1.44–7.82); perception of poor or fair health (OR 2.84, 95% CI 1.28–6.27); older age and perception of risk to self (increasing with the number of events reported, *p*=0.04). Deploying but not with a parent unit and psychological distress were associated in the partially remitted PTSD when compared to the fully remitted group. The positive and negative likelihood ratios for the factors most highly associated with persistent PTSD indicated they were of marginal value in identifying persistent PTSD. Many factors contribute to the persistence of PTSD but none alone is useful for clinical prediction.

### Longitudinal study on psychological impact and cortisol response of Portuguese military to peace mission deployment to Afghanistan 10:30–10:45

#### L. Sales^1,2^, A. Dias^3,4^, M. Roque^5^ and A. Furet^5^

^1^Military Hospital of Coimbra, Coimbra, Portugal; ^2^Centro de Trauma, CES, Coimbra, Portugal; ^3^Utrecht University, Faculty of Social Sciences, Utrecht, The Netherlands; ^4^Centro de Trauma, CES, Coimbra, Portugal; ^5^Military Hospital of Coimbra, Coimbra, Portugal

*Background*: Biomarkers research is an internationally recognized field of interest. Cortisol is one of the most studied, namely within the trauma-focused studies. Despite military staff receiving special training to cope with stressful conditions, deployment and its surroundings may represent an additional stressful task. However, specific previous vulnerabilities may impair their ability to cope adequately with the situation. Considering this scenario, we tried to investigate longitudinally the changes in cortisol response and psychological functioning that occur with the deployment exposure. *Goals*: This study analyses psychological dimensions and salivary cortisol variation in a group of Portuguese military staff that have been deployed to a peace mission in Afghanistan during six months. *Method*: Sixteen male military were assessed in four different moments: before deployment; during the mission; early; and later on after deployment. Military filled psychological scales (Childhood Trauma Questionnaire; Brief Symptom Inventory; Post Traumatic Diagnostic Scale; Impact Event Scale- revised) and collect salivary samples, in three different moments—after awake; half an hour after awake; around 4.00 pm. Statistical analyses such as mean differences and regression analysis will be used for the data interpretation. *Results*: Data are still in analysis. However, we expect to confirm the hypothesis that deployment may increase the risk for PTSD and for psychological symptoms, namely in subjects with previous vulnerabilities such us childhood trauma exposure and atypical cortisol responses. *Proposed discussion*: If the data confirm the tested hypotheses, selection criteria of subjects for risk professions should consider the identified vulnerability factors. The validity of our study is increased by the used of longitudinal methodology. However, better generalizability will be gathered if a matched military control group not exposed to the mission would be assessed as well.

### PTSD and existential concerns of Turkish veterans 10:45–11:00

#### B. Guloglu, O. Karairmak Counseling and Guidance, Bahcesehir University, Istanbul, Turkey

Human-made disasters, mass violence and technological disasters causing threat to life, injury, loss of significant others have destructive impacts on individuals. Military-combat is the most common cause of PTSD among men. The PKK has started guerilla war against Turkish Republic in 1984. Besides attacking security forces, the PKK involved in many violent actions such as suicide bombings, bombing civilians, kidnapping. The PKK has been listed as a terrorist organization by the European Union and the United States. Turkish Armed Forces is the first responder to the PKK terrorist attacks. Army service is compulsory for men after 18 years old in Türkiye. Therefore, male citizens frequently engage in battles with the terrorists. The data were collected from 247 veterans who were seriously injured in battles. PTSD symptoms were screened. Most frequent symptoms were avoidance, re-experiencing, anger, difficulty-concentrating and easily startled. The veterans also answered the open-ended question of what did you lose? 53 of them only listed organ deficiency, the rest both mentioned organ deficiency and other types of loss. While analyzing the data using qualitative methods, six themes emerged: losing-future expectations, reducing-meaning of life, damaging-interpersonal relations, ruining-self-perception, losing-psychological balance and losing-autonomy. Future expectations theme included the words of dreams, springtime of life, future, hope, and hopelessness. Lives, joy of living, view of life were chosen for the theme of reducing meaning of life. My love of life, friends, spouse, brothers in arm, and folks were associated with damaging interpersonal relations. Self-confidence, compassion and pity, ability to make decision, self, self-respect, trusting others were related to ruining self-perception. Losing psychological balance included psychological concerns, furious, angry, hostile, impatient, harsh, unstable, going crazy. Freedoms, dependent, needy, and clingy were related to losing autonomy. The emerged themes were compatible with existential concerns; therefore the results were discussed from the existentialist view.

## ORAL, JUNE 9 HALL GLORIA

## Evidence-based practice on trauma
*
Workshop: To be or not To be - Early interventions following traumatic events*

### To be or not to be: early interventions following traumatic events 8:45–9:45

#### S. Freedman Bar Ilan University, Ramat Gan, Israel

Traumatic events are common occurrences, and whilst cognitive behavioural therapy (CBT) has been shown as an effective treatment for chronic posttraumatic stress disorder (PTSD), controversy still exists regarding early interventions. This workshop will cover the literature regarding early interventions, describing interventions from the first hours post-event, up to those beginning within 1-month post-trauma. Interventions will be demonstrated, both by video and role play. In addition, issues such as optimal timing of interventions, the role of debriefing, the value of early interventions, delivery systems and the management of mass traumatic events will be discussed.

## Evidence-based practice on trauma
*
Symposium: Narrative reconstruction for PTSD - Theory, RCT and changes in narratives*

### Narrative reconstruction for PTSD – theoretical background and outcome study findings 10:00–10:20

#### T. Peri, M. G. Gofman and Z. Vidan Bar Ilan University, Ramat Gan, Israel

The high rates of posttraumatic stress disorder (PTSD) patients who are not helped by current effective psychotherapy methods call for the development of additional new treatment methods (Hoge, 2011; Schynder, 2005). Memory disturbances related to the lack of integration of the traumatic memory within the autobiographical knowledge base are seen as a major factor contributing to intrusion symptoms in PTSD (Brewin, 2011). In this symposium, we propose narrative reconstruction (NR) as a novel module for the treatment of intrusive symptoms and memory disturbances in PTSD patients. NR is a brief and focused intervention (up to 12 sessions) combining elements of cognitive behavioral treatment such as exposure and cognitive restructuring, albeit in a unique way, alongside psychodynamic elements. The goal of NR is to create a cohesive and chronological narrative of the trauma while simultaneously addressing the personal significance of the trauma and integrating it in the patient's autobiographical memory. Theoretical background, treatment description, and potential therapeutic advantages will be discussed. The following presentations will present preliminary results of an ongoing randomized control trial evaluating the efficacy of NR and a study comparing patients’ spontaneous narratives of the trauma before treatment versus after treatment. Presentations will be followed by a discussion of these findings and possible further directions for research.

### Traumatic narratives before and after narrative reconstruction treatment 10:20–10:40

#### Z. Vidan^1^, R. Tuval-Mashiach^1^, L. Jelinek^2^ and T. Peri^1^

^1^Bar Ilan University, Ramat Gan, Israel; ^2^Universitatsklinikum Hamburg-Eppendorf, Hamburg, Germany

Analysis of trauma narratives is a central tool for understanding the characteristics of traumatic memories and their contribution to the development of posttraumatic stress disorder (PTSD). It is assumed that the way in which the trauma story is told provides a window to understanding the structure and organization of the traumatic memory. A large body of research has shown that traumatic memories have different characteristics than other autobiographical memories, that is, they are more fragmented, unorganized, and incoherent in comparison to other autobiographic memories and thus not integrated into the autobiographical memory system. Traumatic memories are also characterized by vivid negative sensorial and emotional content and are accompanied by negative attributions to the self. The current study compared traumatic narratives of 12 patients before and after treatment with narrative reconstruction (NR, Peri & Gofman, in press). A structural analysis was employed to measure narrative disorganization and fragmentation according to guidelines first introduced by Foa et al. (1995) and modified by Halligan et al. (2003) and Jelinek et al., (2009,2010). Preliminary results show a significant increase in narrative organization posttreatment compared to pretreatment (*p*<0.01). The decrease in fragmentation level did not reach significance level yet it was significantly correlated with the reduction in PTSD symptoms as evaluated by the Clinician Administered PTSD Scale (CAPS, *p*<0.05). Qualitative analysis of the formal aspects of the trauma narratives pre- and posttreatment showed that posttreatment narratives had better story line continuity, and fewer memory gaps. Content analysis revealed a stronger sense of control, and a more positive perception of one's self and agency. The results demonstrate the connection between traumatic memory encoding and PTSD, as well as illustrate NR's impact upon traumatic memory.

### Preliminary results of a RCT examining treatment outcome of narrative reconstruction for PTSD 10:40–11:00

#### M. G. Gofman, Z. Vidan and T. Peri Bar Ilan University, Ramat Gan, Israel

A randomized controlled trial (RCT) examining the efficacy of Narrative Reconstruction (NR) is currently in progress. Previously reported preliminary results of an open trial of six post-traumatic stress disorder (PTSD) patients were promising (Peri & Gofman, in press). The effect size for pre-treatment to follow-up changes of PTSD symptoms [Clinician Administered PTSD Scale (CAPS) total score] of 1.66 was slightly higher than that reported in the meta-analysis of psychotherapy for PTSD (1.43) by Bradley et al. (2005). The current study reports initial results of the RCT.16 patients were randomly assigned to two experimental groups: (1) an active-treatment group of eight patients and (2) a minimal intervention wait-list group of eight patients, which served as the control group. All patients met *DSM-IV-TR* criteria for PTSD as ascertained by the CAPS participated in the study. PTSD patients referred to the Community Counseling Service of the Psychology Department at Bar Ilan University who agreed to participate in study were treated by PhD-level interns of the clinic. Treatment lasted for 12 fifty-minute sessions. Patients’ medications were not altered during therapy. Participants were evaluated by trained MA-level psychology students blind to treatment condition, pre-treatment, post-treatment and at 3-month follow-up. Psychometric measures included the CAPS, the self-report post-traumatic diagnostic scale and the beck depression inventory. To date, the effect size for change in PTSD symptoms for the active group versus the wait-list control stands at 1.7, exceeding the average effect size of 1.11 reported by Bradley et al. (2005) for treatment versus wait-list control.

## ORAL, JUNE 9 HALL LADY G

## Effects of trauma on families and children
*
Symposium: Complexities of family adaptation after traumatization. 1. Research findings*

### Psychological and social sequelae of sexual violence: a mixed-method study in Eastern Congo 8:45–9:05

#### A. Verelst Centre for Children in Vulnerable Situations, University Gent, Gent, Belgium

This contribution addresses psychological and social consequences lived by adolescent survivors of sexual violence in Eastern Congo and presents related implications for their psychosocial rehabilitation. Based on a large-scale mixed-method study, the contribution discusses protective and risk factors associated with psychosocial well-being in girls who experienced sexual violence. First, in a school-based study (*n*=1340), self-report questionnaires on posttraumatic stress symptoms, externalising and internalising psychological problems, war-related traumatic events, daily stressors, coping, social support and rape myth acceptance were administered. Furthermore, this multi-method study included a qualitative exploration (*n*=27) of psychological and social consequences of sexual violence. Findings of these intertwined studies show that adolescent victims of sexual violence face a myriad of psychological and social sequelae. Negative social reactions, social support and daily stressors seem to be associated with the psychological impact lived by these adolescent survivors. Implications for psychological treatment of the diverse responses to traumatic stress in adolescent survivors within the specific context of Eastern Congo are formulated.

### The effects of occupations and change of political system to second generation of Lithuanian survivors of political repression 9:05–9:25

#### I. Vaskeliene, D. Gailiene, E. Kazlauskas and N. Grigutyte Faculty of Philosophy, Vilnius University, Vilnius, Lithuania

*Background*: In twentieth-century Lithuania experienced two world wars, Nazi and Soviet occupations. Independence of Lithuania from the last Soviet occupation was restored just in 1990. All the population was effected by the change of political system and implemented assimilation policy from occupation regime. About one-third of Lithuanians directly suffered from Nazi and Soviets repressions. The hypothesis is that second generation of survivors of these political repressions would experience long-lasting psychological effects. This presentation will point out these long-lasting intergenerational effects. *Methods participants:* Transgenerational effects of political repression were analysed in a sample of 145 participants whose mother or father survived Soviet or Nazi political violence. Their results were compared with a sample of 177 participants matched according to socio-demographic characteristics and whose parents did not directly experience political repression, and 66 participants whose parents survived Holocaust experiences. Besides, the parents of the second generation of survivors of political repression participated in the study. Second-generation participants completed questionnaires which assessed subjective experiences of parents’ political trauma, their lifetime trauma experiences, present post-traumatic stress symptoms, sense of hopelessness and coherence. *Results*: The results indicate that second generation of survivors of political repression related their life difficulties during Soviet occupation to parents’ status as survivor, and associated this with well-being, the relationship with their parents, and attitudes. The effects of political change and restoration of independence in the country were also indicated: it stimulated more open communication in families about the experiences of political violence. Present mental health measures between the three second-generation groups revealed one statistically significant difference: post-traumatic hyper-arousal is more intense among second generation of survivors of political repression. The performed path analysis identified intergenerational links of mental health between survivors and their offspring.

### The absent father? Quantity and quality of father involvement in a refugee sample 9:25–9:45

#### E. Van Ee Foundation Arq, Diemen, The Netherlands

Parental traumatisation has been proposed as a risk factor for child development, but the mechanisms involved are poorly understood. Despite increased attention on the role of fathers within families there is still a dearth of studies on the impact of trauma on father involvement. The presented study investigated the quantity and quality of father involvement and the influence of post-traumatic stress on the quality of involvement in a refugee and asylum seeker population. Eighty refugees and asylum seekers and their young children (aged 18–42 months) were recruited. Measures included assessment of parental trauma (Harvard Trauma Questionnaire), quantity and quality of involvement (quantity of care-giving and Emotional Availability Scales), and perception of the father–child relationship (interview). This presentation will provide unique data of structured and thorough observations of parent-child interactions among refugees often severely traumatized by war. The results show that fathers were less involved in care-giving tasks and play activities than mothers. No parental gender differences were found on each of the Emotional Availability Scales. Traumatic stress symptoms negatively affected the perception and the actual quality of parent-child interaction (sensitivity, structuring, non-hostility). Most fathers acknowledged the negative impact of post-traumatic stress on the relationship with their child and desired an improvement as the relationship with their child is of importance to them. Still, almost all fathers described the relationship with their child as good. Despite the impact of post-traumatic stress, refugee fathers have a certain involvement within the lives of their children. As the quality of father-involvement is of importance to the development of the child, traumatized fathers are as much in need of clinical intervention as mothers.

## Effects of trauma on families and children
*
Symposium: Complexities of family adaptation after traumatization. 2. Clinical expertise*

### The individual's psychic trauma as group transition 10:00–10:15

#### T. Toscani Istituto di Terapia Familiare di Bologna (ITFB), Bologna, Italy

In the past 13 years, the Institute of Family Therapy in Bologna,founded in 1996 (associated to SITF, AIMS, EFTA and affiliated to SiSST), has dealt mainly with clinical manifestations of simple and complex post-traumatic stress disorder (PTSD) when treating individual, families and couples. Within our trauma team, we apply diverse approaches and methodologies for diagnosis and therapy, combining individual trauma treatment and family therapy based on relational-systemic framework. In our work with families, we use an integrated model, employing the contextual diagnosis, systemic-relational approach, combined with insights from psycho-traumatology and the new validated work techniques on traumatic memories. The interventions are focused connecting the event to the post-traumatic response and to the pre-traumatic personality. Bonds and family relationships can be a resource or a hindrance in the healing of psychic trauma. The diagnosis and the treatment of simple and complex post-traumatic disorder of the individual is contextualized within his/hers family, couple and social significant relationships. The person's suffering and his/hers treatment pathway becomes a choral event within the relationship, which returns responsibility, rights and hopesto each actor. This contribution to the workshop will with just a few clinical slides, illuminate how within very different treatment pathways the clinical work with family and parental network constitutes an important resource for the diagnosis and treatment of psychic trauma. Considering psychic trauma an individual response as well as a group response implies being conscious that to treat trauma within significant relationships opens up the hope of transmitting to future generations that from painful events of one's own history, one can evolve.

### Therapeutic dialogue with refugee families: stories of trauma, stories of culture, stories of an encounter between home and host societies 10:15–10:30

#### L. De Haene^1^ and P. Rober^2^

^1^Faculty of Psychology and Educational Sciences, K.U.Leuven, Belgium; ^2^Institute for Family and Sexuality Studies, K.U.Leuven, Belgium

This workshop engages in a participative exploration of relational complexities involved in therapeutic work with refugees. Building on clinical case material, we address how the ongoing dialogue between refugee clients and clinician implies complex relational processes of negotiating remembrance and forgetfulness within the therapeutic space. The workshop explores different relational and social meanings from which to understand and engage in this balancing movement between silencing and disclosure, remembering and forgetting in the therapeutic encounter. First, we address how silence and disclosure in refugees’ stories of collective violence and loss echo the dual imperative to both forget and witness that is invoked by man-made atrocity and that resonates in community, family, and individual responses to refugee trauma. Furthermore, we address how silencing communication strategies may be rooted in clients’ cultural worlds and how the encounter with cultural alterity may invite an open negotiation of divergent universes of meaning and action. Lastly, we explore how relational transactions of silencing and disclosure may also touch upon intricate power disparities between refugee clients and clinician. This imbalance between the therapist's social position and refugee clients’ isolation calls for an attentive reflection on how negotiating remembrance may be experienced as imposing and reiterating inequality, while equally indicating how refugees’ stories as told in the clinical context may be marked by their broader social context that silences and denies these stories of dislocation. In relating to these different meanings of remembrance and forgetfulness, this workshop will primarily explore ways of respectfully engaging their relational transactions into therapeutic conversation, opening new spaces of dialogue and expressing the therapist's willingness to accept co-authorship for what is said, not said, and not feasible to say in a clinical space that is inevitably located at the nexus of subjective and sociopolitical meaning.

### Mind the babies: an early intervention method that focuses on strengthening the bond between infants and their traumatized mothers 10:30–10:45

#### I. Hein and A. Jasperse Foundation Centrum ‘45, Diemen, The Netherlands

This workshop will introduce a newly implemented Infant Mental Health group treatment that focuses on the relationship between babies (children under 1 year) and their parents suffering from post-traumatic stress disorder (PTSD), depression and/or anxiety. Patients who participate are refugees or victims of human traffic, without a stable living situation. Most children are born after involuntary sexual contact. The ongoing stress and the severe complaints create high risk for developing attachment problems. We work according to the principles of Infant Mental Health (Slade et al.) combined with the short-term group treatment for anxious/depressed mothers and their babies as developed by Grinsven et al. The approach is based on knowledge of the psychodynamic theory of early development of children, development of parenthood, and impact of psychiatric disorders on the parent–infant interaction. The group treatment includes both preventive and curative components. The theoretical background and the practical elaboration will be explained and illustrated with video segments. The first results and suggestions for outcome monitoring will be discussed.

### The power of multifamily therapy with traumatized parents and their children 10:45–11:00

#### T. Mooren^1^ and J. Bala^2^

^1^Foundation Centrum ‘45, Oegstgeest, The Netherlands; ^2^Foundation Centrum ‘45, Diemen, The Netherlands

In this contribution to the workshop, we will present and illustrate characteristics and rationales of the Mentalization-based Multifamily Therapy (MFT) for traumatized asylum seeker- and refugee-families who has been developed in Centrum ‘45, the Netherlands. We have gained about 10 years of expertise by now in working with MFT. MFT is particularly effective in increasing parent–child interaction and reestablishing parental skills and competencies such as showing affection, mentalizing the child's needs, structuring and guiding, sharing pleasurable activities in a setting with playful atmosphere. These competencies related to the emotional availability are exactly those capacities that have been lost or undermined for parents with post-traumatic stress disorder (PTSD) and comorbid complaints in the aftermath of severe violence and/or migration. This presentation will illuminate the intervention-principles by video segments and examples of interventions and activities for groups. Outcomes of assessments of parent–child interactions (using the Emotional Availability Scales Biringen, 2008) will be discussed.

## ORAL, JUNE 9 HALL LIZ

## Morning
*
Symposium: Traumatic events and personality disorders*

### Traumatic events and personality disorders 8:45–9:00

#### J. Luigi Dipartimento di Psichiatria, Universitá Cattolica del Sacro Cuore, Rome, Italy

Although guessable, a pathogenetic relationship between stressful or traumatic events, especially in childhood, and the development of at least some personality disorders, in literature there are no systematic studies have investigated the effect of these events on patients with personality disorders. It is well known that the adaptive response to traumatic or stressful events can become dysfunctional for the intensity and for the duration of requests and for the characteristics of the subject, and on the other hand, personality is one of the factors that most influence the vulnerability to stress. Thus, the presence of a personality disorder can affect and change the perception, meaning, and reactions to stressful events. In fact, some personality disorders (paranoid, avoidant, dependent, borderline) are significantly associated with the development of a possible PTSD. In addition, in view of the relationship between environment and genotype, individuals with personality disorders tend to be exposed to more stressful situations, which in turn change the structure of personality. Finally, established that the presence of early traumatic experiences is one of the factors behind the development of these disorders.

### Trauma and disruption in different mental operations 9:00–9:15

#### A. Mandese and M. Petrollini Scuola dell'Accademia di Psicoterapia Psicoanalitica (SAPP), Rome, Italy

The power of the different situations disorganizzativa traumatogenic is addressed in this work in key psychodynamics. Paying particular attention to the case when the film narrative and life is blocked and turned into a still picture film, consisted of the associative mode of thought gives way to a sort of “assembly” with a major impact on the emotions and behaviors. Of course, all these happen in very different ways depending on the developmental level of psychic functioning, for which space is reserved for the description of how to manage the disorganization that characterize the personality belonging to the three major areas: psychotic, neurotic, and organization limit.

### Relationship between traumatic events and structure of personality in the genesis of PTSD 9:15–9:30

#### P. Cimmino Private practice, Rome, Italy

In this work, the criteria for defining the post traumatic stress disorder (PTSD) will be presented schematically and the changes in cognitive, emotional, and psychological levels. The objective is to consider some risk factors that can prepare the development of PTSD.

In that regard two key points will be investigated:

1. Childhood trauma and disruption attachment.
2. Dimension dissociative disorders some personality and disorder post-traumatic stress.

It is called between the differeziazione PTSD the first and second type type, listing studies and models of different authors who have tried to consider:

1. The vicious circle that the interpretation established between traumatic event and chronic PTSD.
2. The etiological factors affecting the transition from the stress disorder resulting in trauma.

### Therapeutic work when the defense turns trauma: a case report 9:30–9:45

#### D. Laghi Scuola dell’Accademia della Psicoterapia Psicoanalitica, Viganello, Switzerland

The personal ability to cope up with traumatic situations and process varies widely from individual to individual, not only in terms, we may say generally, the operation of the underlying personality, but also the peculiar position that the painful experiences or catastrophic going to play within a system of thought. If we assume that the psyche is built around the mental representations and the construction of internal working models, even traumatic experiences will be featured influential family histories and, in some cases, become clothes to wear, ways of life, part of the basis identity, even. All the energy used to contain and exclude contact with their painful experiences will be subtracted from the ability to associate, mentalizing, be introspective spontaneously and truthfully. The therapeutic work will take advantage of every communication channel, to trace elements that reveal what cannot be told in words, with the aim of restoring those logical and emotional connections between events and meanings of events in the mind traumatized they had to stop. The clinical case presented provides insights on building the therapeutic alliance and the ability of the therapeutic relationship to hold, organize, and edit the trauma giving a different meaning in history and family life of the subject.

## *Trauma and Los*s

### Loss of employment: when personal resources are not enough. The contribution of EMDR 10.00–10.15

#### Elisa Faretta, Tiziana Agazzi and Valentina Zambon Centro Studi P.I.I.E.C., Milan, Italy

“The loss of employment” is a very important topical issue in this period of economic crisis, especially because of the impact that this stressful event has on psychological and emotional aspects. The dismissal could be considered as a critical and “traumatic” event that could destabilize and lead to the development of a sense of powerlessness, frustration, and maladjustment, and then to an adjustment disorder or PTSD. With EMDR, an evidence-based treatment for PTSD, we help patients change negative cognitions about themselves and reprocess dysfunctional emotions related to critical events such as job loss. By following the model of “past–present–future”, it is possible to promote the processing of memories connected to the most stressful moments, the situation in the present that can reactivate the trauma of those moments, and finally preparing them to face the future situations in a more adaptive and positive manner. Several cases will be reported.

### The wounded territory: psychopolitic intervention strategy and resilience 10.15–10.30

#### Ilaria Cinieri Istituto di Specializzazione in Psicoterapia Umanistico-Bioenergetico Psicoumanitas S.r.l.Taranto, Taranto, Puglia, Italy

The psychopolitical model, founded in 1976 by thoughts and feelings of Luigi De Marchi, was presented as a dynamic analysis of large social processes. Using its foundations we have checked the possibility of individual and social empowerment to bring the city to a state of well-being in building social holding.

Taranto was the home city of our research from 2008 to 2012. Developing and updating the application of the analytical technique of De Marchi, a real intervention strategy was found, which proposed — starting from the ‘Social Outing Project’—new ideal models of ergonomic policy and right to well-being in response to environmental traumas. The city was declared in financial difficulties (2006) and in a state of environmental disaster, and sees in the poisons of the ILVA steel factory the main causes of the increase of malignant diseases, cardiovascular, and respiratory mortality with a consequent increase of the pediatric one (30% up to 400%). Therefore, it is a sadly privileged observatory with regards to the modality and timing of activation and organization of resources aimed at the overall resilience. Through a methodology that continues his experiments within cooperative mediation, the work reflects the idea of transformation and the willingness to share (Social Self Help Group). These seem to be the main needs accompanied by feelings of loneliness and abandonment recorded in the listening phase. Through several layers and social contexts, a representative sample of individual experiences, generational, cultural, and cross-cultural as well as political-administrative was collected. Hence, what techniques will we have to reinterpret, learn, practice, and supervise, so that, they can be useful in enabling a resilient social/environmental/individual that facilitates the action in the real world, where even the generational proximity becomes a resource? The S.O.T. method used allows one to sense the process of restructuring the social identity and of those who are living in a wounded territory.

**References**

De Marchi, L. (1984). *Scimmietta Ti Amo- da Neanderthal agli scenari atomici*. Edizioni Longanesi, Milano (IT).

Gordinier, J. (2008). *X saves the World*, Penguin. USA.

Lo Iacono, A., Milazzo, P. (2007). *La sala degli specchi: comunicazione e psicologia gruppale*. Franco Angeli, Milano (IT).

### The influence of psychosocial factors on human behaviour in emergency 10.30–10.45

#### Elisa Saccinto^1,2^, Carles Pérez-Testor^1^ and Luca Pietrantoni^2^

^1^Blanquerna Facultat de Psicologia, Ciències de l'Educació i de l'Esport, Universitat Ramon Llull, Barcelona, Spain; ^2^Dipartimento di Psicologia, Alma Mater Studiorum Universitá di Bologna, Bologna, Italia

The aim of this study is to explore the effect of some psychosocial factors on human behaviour during an emergency situation. In agreement with the observations of the theory of social attachment in disasters (Mawson 2005, 2007), people in danger experience feelings of attachment for familiar persons and places. These feelings have a reassuring effect, reducing or delaying the behaviour of evacuation. Participants were 173 survivors of several emergency situations: 42.4% were men and 57.6% were women with mean age 32.52 years (SD = 13.68). Results evidenced a negative association between being with familiar people and delay in starting the evacuation, whereas the presence of familiar people or being at home during the danger situation were not significantly associated with evacuation. Findings also highlighted significant associations between participants’ emotional response and place: participants who were at home during the danger situation experienced more panic symptoms, distress, and perception of threat in the different moments of the evacuation in comparison with those who were in public buildings. Finally, it was concluded that the presence of familiar people had an influence on the level of distress during emergency situation. Here, we discuss some implications of these results for prevention and education programs on human behaviour during emergencies and for improving citizens’ preparedness.

**References**

Mawson, A. R. (2005). Understanding mass panic and other collective responses to threat and disaster. *Psychiatry, 68*, 95–113.

Mawson, A. R. (2007). *Mass Panic and Social Attachment. The Dynamics of Human Behaviour*. Brookfield (VT): Ashgate Publishers (Eds).

### Trauma and foreignness: a model of clinical intervention 10.45–11.00

#### Chiara Marangio Department of Human Sciences, University of Urbino, Urbino, Italy

This paper focuses on the clinical relationship with foreign entities who report the aspects of post-traumatic stress. Starting from the discussion of the concepts of culture and context between modern and post-modern paradigms, a general model of clinical intervention involving a form of negotiation setting based on extraneousness and contingency is proposed. Context and culture do not respond to universal categories and limited space-time frames, but they are the way in which the subject means the world. This semiotic clinical disposal represents a criterion of knowledge of the individual configurations and also an instrument of emerging post-traumatic forms. The clinician knows the way in which the individual gives meaning to their experience and the form that the trauma takes on its basic operation. The negotiating prospect moves away from the idea of the person as a suffering carrier or a victim and connotes the person as a builder of experience, so with agency. It is possible to intercept the personal resources to orient a contingency-based intervention that promotes a process of co-construction of meaning, a product of the relationship between the actors. This generative aspect acts on the traumatic forms of the agent, opening a prospect of development and change. This psychological approach to the crisis is not like the phenomenal, psychiatric and ordinal categories, but in the sense that the subject gives the experience, even when it is dysfunctional for their existence. The object of the relation are the relational process and the person in its complexity, not only in the sense of traumatized and/or a stranger but also as the conditions of partial state and nonexistence.

**References**

Carli, R., Paniccia, R. M. (2003). Analysis of the demand. Theory and Technique intervention in clinical psychology. Il Mulino Edition.

Salvatore, S. (2004). Unconscious and speech. The unconscious as a discourse. In M. B. Ligorio (Eds.), *Psychology and Culture: Contexts, Identities and Interventions* (pp. 129–158). Rome, Carlo Amore Edition.

Venuleo, C. (2008). What is the nature of idiographic date? In *Yearbook of Idiographic Science: Vol. 1/2008* (pp. 273–283). Rome, Firera & Liuzzo Publishing.

### The missed appointments and the tears of Niobe: the pain of not occurrence 11.00–11.15

#### Antonio De Luca Ministero della Giustizia, Cosenza, Italy

When something breaks the lived imposing itself as traumatic, it also exposes a prospect invisible: it is the not the occurrence of something else. It is the existential missed appointment: what would have happened but did not happen. Suspended along the history, the sudden death of the beloved person not only digs deep furrows in the folds of the soul. It does not throws only a shadow acute on track for what happened, it don't petrify places and the beat of time and hope while everything is wrapped in the area of rarefied suspension. It also requires invisible sequences of what, from that moment onwards, cannot be achieved together. All those moments are important, both in daily life and in the uniqueness of an event. It is then that appears as the pain of not occurrence, laying a trap for karst of absence of someone/something. In the petrification of Niobe, only tears appear in a frozen time being also source capable of quenching thirst lives suspended between the memories on the pile of rubble not becoming ruins, in spite of all. What remains after an emotional destruction is that fragment of the events together with what didn't happened. What suffering then for what didn't happen? Which “symptoms” and “processing” of that never happened? Will be discussed clinical situations and affects related to “missed appointments” and the possible therapy.

**References**

De Luca, A. (2000). Gli appuntamenti mancati e lo sguardo di Sisifo. *Comprendre, 10*, 89–99.

De Luca, A. (2011). *Tra le rovine dell'esistenza. Sofferenza Psicoterapia Ripresa* (Intr. di B. Callieri, Pres. di G. Di Petta). Roma: Edizioni Universitarie Romane.

De Luca, A. (in press). *La nostalgia infinita dell’altro e di sé: gli appuntamenti mancati e il dolore del non accadimento*, in A. De Luca, A. M. Pezzella (edd.), *Con i tuoi occhi. Sull'intersoggettività*. Milano: Mimesis.

## ORAL, JUNE 9 HALL SAVOIA

## The spectrum of trauma-related disorders
*
Workshop: What every clinician should know about dissociative identity disorder - Diagnostic and therapeutic considerations*

### What every clinician should know about dissociative identity disorder: diagnostic and therapeutic considerations 8:45–9:45

#### V. Sar Department of Psychiatry, Istanbul Faculty of Medicine, Istanbul University, Istanbul, Turkey

Dissociative identity disorder (DID) affects 1.1–1.5% of the general population. These rates are higher in psychiatric inpatient (5.4%), outpatient (2–2.5%), emergency outpatient (14.0%), and adolescent outpatient (16.4%) units. There are certain risk groups such as chemical substance users (5.8%) and women in prostitution (18%) (Sar, 2011). Subtreshold DID is much more prevalent than the full picture. Among all psychiatric disorders, DID is the one with highest frequencies of childhood psychological trauma at the antecedents of the condition. DID is the only psychopathology where no specific (“anti-dissociative”) effect can be obtained by drug treatment. While DID can be treated succesfully by intensive outpatient psychotherapy, psychiatric and social consequences of untreated DID may be devastating (Mueller-Pfeiffer et al., 2012). Systematic standardized clinical assessment has to evaluate basic dimensions of dissociative psychopathology: amnesia, depersonalization, derealization, identity confusion, and identity alteration. However, to screen DID in daily practice succesfully, one has to know the secondary features of the disorder: affect dysregulation, chronic depression, somatic complaints, interpersonal relationship difficulties, concentration problems, and persistant suicidality are among them. Psychotherapy of DID is based on the three-stage trauma resolution. The most frequently encountered pitfall the denial of the diagnosis by the therapist and lack of basic understanding about dissociation as a clinical condition. With its comprehensive content, this workshop is aimed at an up to date presentation of DID both for those who are less familiar with the condition as well as for clinicians and researchers who are experienced in diagnosis and treatment of dissociative disorders.

References

Mueller-Pfeiffer, C., Rufibach, K., Perron, N., Wyss, D., Kuenzler, C., Prezewowsky, C., et al. (2012). Global functioning and disability in dissociative disorders. *Psychiatry Research*, *200*(2–3), 475–481.

Sar, V. (2011). Epidemiology of dissociative disorders: An overview. *Epidemiology Research International*, *2011*, 8. doi: 10.1155/2011/404538

## The spectrum of trauma-related disorders
*
Workshop: Violence and trauma - A multimodal violence specific psychotherapy*

### Violence and trauma—a multimodal violence specific psychotherapy 10:00–11:15

#### B. Loemo, I. R. Askeland, J. Strandmoen, T. Heir and O. A. Tjersland Norwegian Centre of Violence and Traumatic Stress Studies, Oslo, Norway

*Aims*: The majority of treatment programs for men using intimate partner violence (IPV) have their main focus on attitudinal and behavioral change. The aim of this workshop is to present empirical and clinical support for integrating a trauma focus and general psychological knowledge in IPV treatment programs. *Methods*: Traumatic Experiences Checklist and the Mini International Neuropsychiatric Interview (MINI 6.0.0) were administered in a pretreatment clinical evaluation of 192 men who voluntarily attended treatment for IPV. *Results*: The majority of the men (76.2%) reported potential traumatic experiences in their family of origin. Six out of 10 (61.8%) had experienced physical abuse from their parents or older siblings. The majority of the men (70.1%) fulfilled the diagnostic criteria for at least one psychiatric diagnosis, measured by MINI. Nearly 2 out of 10 (18.5%) qualified for a posttraumatic stress disorder diagnosis. *Discussion*: Associations between trauma, diagnoses, and violence will be discussed. Our findings point to the need for interventions based on a broad spectrum of psychological theories and interventions in addition to a cognitive behavioral approach. In addition to being a behavioral problem, IPV can be understood as a trauma-related disorder. To illustrate clinical pathways we will present two single cases. These clinical cases illustrate how to work trauma focused within the frame of IPV treatment.

## ORAL, JUNE 9 HALL STUART TUDOR

## Open Papers: Children and young people I

### Forgiveness and spirituality in childhood trauma 08:45–09:00

#### B. Guloglu and O. Karairmak Counseling and Guidance, Bahcesehir University, Istanbul, Turkey

Childhood trauma, including abuse and neglect may cause long-term physical and mental health problems like posttraumatic stress disorders and depression, and also issues in social functioning like homicidal ideation, legal problems, sexual and running away behaviors into adulthood. After a person has incurred any kind of transgression, forgiveness is an important factor for psychological well-being. Forgiveness refers to consciously and willingly fostering positive emotions such as empathy, compassion, and affection instead of negative emotions such as anger, resentment, and hostility toward an offender. Forgiving people tend to have more emotional stability, hope and self-esteem. However, childhood trauma experience can make difficult for victims to cope with anger and forgive the transgressor. Spirituality is associated with forgiveness. People who consider themselves as spiritual tend to value forgiveness highly than less spiritual people. As a belief in a power apart from one's own existence, a sense of connectedness to self, others, nature or God, a quest for wholeness, a search for hope and harmony, spirituality is an essential factor for meaning and purpose in life. People with trauma history come to the term that the world can be unsafe, unjust, and meaningless. Hence, the aim of this study is to investigate the role of childhood trauma on forgiveness and relationship. Childhood Trauma Questionnaire, Heartland Forgiveness Scale, and Spirituality Scale were administered to 527 Turkish university students. The results of MANOVA that were applied to Childhood Trauma Questionnaire scores yielded a significant overall main effect of forgiveness [Wilks’Λ=.920, *F*(1, 526)=21.788, *p*<.001, *η* ^*2*^=.059) and spirituality [Wilks’Λ=.920, *F*(1, 526)=32.811, *p*<.001, *η* ^2^=.040). University students who have childhood trauma have lower level of forgiveness and spirituality than students who weren't induced to trauma in childhood.

### Secondary victims of rape 9:00–9:15

#### A. Elklit^1^, R. Bak^2^ and D. Christiansen^3^

^1^National Center for Psychotraumatology, University of Southern Denmark, Odense, Denmark; ^2^Center for Rape Victims, Aarhus University Hospital, Aarhus, Denmark; ^3^Department of Psychology, Aarhus University, Aarhus, Denmark

Rape is often a very traumatic experience, which affects not only the primary victim (PV) but also his/her significant others. Studies on secondary victims of rape are few and have almost exclusively studied male partners of female rape victims. The present study examined the impact of rape on 107 secondary victims, including family members, partners, and friends of male and female rape victims. We found that many respondents found it difficult to support the PV, and that their relationship with the PV was often affected by the assault. Furthermore, the sample showed significant levels of traumatisation, and it was estimated that approximately one quarter of the respondents suffered from PTSD. Degree of traumatisation was associated with a more recent assault, higher efforts to support the PV, recurrent thoughts about having been able to prevent the assault, a lack of social support for the respondent, and feeling let down by others. The respondents were generally interested in friend, family, and partner focused interventions, particularly in receiving education about how best to support a rape victim.

### Second generation,to leave a childhood 9:15–9:30

#### E. Klefbeck Red Cross Center for tortured refugees, Stockholm, Sweden

At the treatment center we meet tortured refugees and their relatives, among them many young adults about to handle their on life and future. Many young adults ask for help in crises and describe that they now, on the footstep to independent life start to reflect on how the fate of their parents have influenced their childhood. That trauma can follow into the lifes of the next generation. Those young adults often describe how they, as children, carried a responsibility that nobody did perceive. The silence, nobody did go near to the traumatic history, with the good intention to protect the children, but it did give space to a lot of images. Often children have experienced their parents unexpected reactions in situations that recalled earlier traumatic events, without understanding what was happening. In this presentation I want to convey the experiences that those young man and women are describing and the internal processing that it takes to be free.

### Promethean trauma in genomic disease families 9:30–9:45

#### E. Acquarini Dipartimento di Scienze dell'Uomo, Universitá degli Studi di Urbino, Urbino, Italy

The emotional impact of a rare metabolic syndrome (Niemann-Pick disease) is a potentially traumatizing mixture at both individual and family level. *Promethean trauma* involves parental expectations of an unhealthy genetic transmission to the child: the traumatic impact of this kind of diagnosis dismantles aspects of parental generativity falling into paradoxical one and causes the loss both of ideal child and a shared future (A-MNP, B-MNP). Individually, can be observed a complex interplay of negative emotions (i.e., shame, guilt, fear, anger, helplessness) in conjunction with patterns of resilience that can affect each family member exposed to the traumatic context. Furthermore, the polymorphic symptoms may favor the development of psychiatric symptoms (C1-MNP) prior to neurological onset delaying a proper diagnosis. In the long term the reactions become more organized at temporally settled in more differentiated actions and feelings that can be prodromic to altered states of consciousness and psychic fragmentation where each psychic fragment suffers on its own. Individual and familiar resilience has to activate the shared resources to promote an adjustment process that can delay positively the disease progression. It is crucial to reflect on the quality of experiences that MNP patient lives in their family to pull out from this *nobody time* and to help the maintenance of meanings and limit the paradoxical traumatic vacuum.

References

Ferenczi, S. (1930). *Note e frammenti. Trauma e aspirazione alla guarigione*. In: *Opere* vol. IV: 1927–1933, Raffaello Cortina Editore, Milano.

Van der Kolk, B. A. (2005). Developmental trauma disorder. Toward a rational diagnosis for children with complex trauma histories. *Psychiatric Annals*, *35*(5), 401–410.

Yanjanin, N. M., Vélez, J. I., Gropman, A. et al. (2010). Linear progression independent of age onset in Niemann-Pick disease type C. *American Journal of Medical Genetics Part B: Neuropsychatric Genetics*, 153B (1), 132–140.

## Open Papers: Children and young people II

### Prior victimization, traumatic birth experience of mothers and the quality of attachment of their young children 10:00–10:15

#### O. Bogolyubova and N. Pleshkova St. Petersburg State University, St. Petersburg, Russia

The goal of this explorative research project was to explore possible connections between prior victimization, traumatic birth experiences of mothers and attachment of their infants. Ten women with traumatic birth experience (aged 22–33 years old) and their children (aged 11–16 months) took part in the study. The following methods were used: (a) The Strange Situation Procedure was used for attachment classification. The videotaped procedures were classified according to criteria of M. Ainsworth and P. Crittenden (Crittenden, 2002); (b) Birth Experiences Interview with consequent analysis of trauma narratives according to the method described by Foa (1995); (c) questionnaires for the assessment of demographics, obstetric history, symptoms of postnatal PTSD (PPQ, Callahan, 2006) and history of lifetime trauma and victimization. The results demonstrated that the mothers, who took part in the study, experienced a wide range of trauma and victimization in childhood and adult life prior to childbirth. It must be noted that women with a history of sexual trauma were more likely to report high levels of postnatal PTSD symptoms. Of all the study participants, 40% demonstrated clinical level scores on PPQ (measure of postnatal PTSD). The analysis of trauma narratives demonstrated high levels of traumatic disorganization. It is interesting to note that Dissociative symptoms in the narratives were present exclusively in the interviews of women with sexual trauma history. The study results demonstrated that 80% of the children in the sample have complex patterns of attachment. The analysis of attachment patterns in connection with the mother's traumatic birth experiences revealed that complex patterns, combining two types of strategy and/or depressive state are observed more frequently in children, whose mothers have had traumatic birth.

### Physical, verbal, and relational revictimization among adolescent girls with histories of maltreatment: the mediating role of PTSD 10:15–10:30

#### W. Auslander^1^, T. Edmond^1^, S. Tlapek^1^, J. Threlfall^1^ and J. Dunn^2^

^1^Washington University School of Social Work, St Louis, USA; ^2^University of Missouri-St Louis, St Louis, MO, USA

Childhood physical and sexual abuse has been linked to revictimization, and girls may be more vulnerable than boys. Few studies have examined the association between child maltreatment types and types of revictimization, and the potential pathways involved. In the present study, the following questions were addressed: (1) What is the association of childhood physical and sexual abuse and revictimization (physical, verbal, and relational) among adolescent girls, and (2) Does PTSD mediate this relationship? The study utilized baseline data from a trauma-focused CBT study that included 150 adolescent girls, ages 12–18 years old (mean age=14.9). The sample was primarily youths of color (83%), and 17% white. Structured interviews included: (1) Frequency of experiencing physical, verbal, and relational aggression (last 3 months); (2) PTSD symptoms; (3) Physical Abuse, Sexual Abuse; and (4) Demographics and other control variables (age, race, living situation, home instability, and service use). Results indicated that 91% of the girls experienced some form of victimization in the last 3 months. Physical abuse was significantly associated with relational victimization (r=.17, *p*<.05). Likewise, girls who experienced sexual abuse reported more verbal and relational victimization (r=.17, *p* <.05) than those who did not experience sexual abuse. Fifty-one percent of the girls endorsed PTSD symptoms in the clinical range. Results showed that higher levels of PTSD were significantly associated with all types of victimization and abuse. Bootstrapped confidence intervals confirmed the significant mediating (indirect) effect of PTSD between sexual abuse and verbal and relational victimization (*p*<.05). PTSD did not mediate the relationship between physical abuse and victimization. A pathway by which sexual abuse influences adolescent revictimization is through PTSD symptoms. PTSD can increase vulnerability to multiple types of victimization. Results suggest that trauma treatment to reduce PTSD symptoms may be an important strategy for preventing revictimization in this population.

### Migration and aggressive behavior in children of traumatized parents 10:30–10:45

#### J. Mueller and N. Morina Department of Psychiatry, University Hospital Zurich, Zurich, Switzerland

*Background*: Research shows correlations between posttraumatic stress disorder (PTSD) and aggressive behavior. It is unclear, however, if forced migration into exile adds on these problems. Aim of our study was to compare aggressive behavior of children of parents traumatized by the Kosovo war who live in exile (Switzerland) with the same behavior of children still living in their home country. *Methods*: We assessed *N*=150 pairs of children and at least one of their parents, *N*=114 of those were still living in Kosovo. Trained interviewers conducted the assessment that included traumatic event types, posttraumatic stress disorder (UCLA PTSD INDEX for DSM-IV and Posttraumatic Diagnostic Scale), aggression (The Aggression Questionnaire) and the children's social behavior (Strengths and Difficulties Questionnaire children version). *Results*: Children of the Swiss sample indicated significantly more traumatic event types as those of the Kosovar sample. However, children of the Kosovar sample showed higher PTSD symptom severity as well as higher aggression than the Swiss sample. Children of both samples did not differ regarding their social behavior. Generally, PTSD symptom severity was correlated with aggression and social behavior, respectively. *Discussion*: The rates of posttraumatic stress disorder in Kosovar adults and their children are still high 11 years after the Kosovo war. According to previous studies, aggression was correlated with PTSD symptom severity. Possibly, the fact of living in a postconflict country is more stressful than having to adapt to a new culture of a safe exile, leading to a higher vulnerability of individuals living under the first condition.

### Terror, trauma, and resilience in the lives of homeless and prostituted street youth: implications for services and community response 11:00–11:15

#### L. Williams University of Massachusetts Lowell, Lowell, MA, USA

This paper will report results from analysis of the narratives of homeless, runaway, and sexually victimized (prostituted and trafficked) youth. This field-based, qualitative study of 61 teens (14–19 years of age) focused on learning from voices of the youth and understanding their lived experiences. Funded by the US Department of Justice, youth were interviewed in two large urban areas: Boston, Massachusetts, and Washington, DC. Findings will be presented on trauma experienced by these youth over the life course (including abandonment, sexual exploitation, and physical violence) and the trauma services and other support resources needed for impoverished and traumatized youth who are not living in “traditional” families. Analyses of the interview data provide evidence of the trauma suffered by the youth, their patterns of internal migration, and their survival-based coping skills all of which suggest a need for the development of meaningful partnerships between street youth and a network of social and mental health service providers. This work has applicability to young males and females who are runaway, homeless, or internal migrants as well as children and adolescents who are trafficked domestically or who cross-national borders to escape conflict in their homes and communities. The paper will provide information to practitioners and community services to increase the safety and well-being of street youth and respond to the trauma they have suffered.

## ORAL, JUNE 9 HALL SYDNEY

## Open Papers: Effects of abuse and violence

### Looking for community interventions for adults exposed to childhood maltreatment 8:45–9:00

#### A. Dias^1,2^, L. Sales^2,3^, J. Mendes^2,4^ and R. Kleber^1,5^

^1^Faculty of Social Sciences, Utrecht University, Utrecht, The Netherlands; ^2^Centro de Trauma, CES, Coimbra, Portugal; ^3^Coimbra Military Hospital, Coimbra, Portugal ^4^Faculty of Economics, Coimbra University, Coimbra, Portugal; ^5^ArQ Research Foundation, The Netherlands

*Background*: Childhood maltreatment is considered a huge problem in both developed and developing countries. Although strong efforts are being made for the prevention in children, a large group of affected subjects is not identified by official services, and does not engage in any intervention. Despite the resilience of some subjects, most of them reach adulthood with an increased risk for impaired mental, physical, and social health. Depression, suicide, substance abuse, and sexual risk behavior are frequently stressed consequences. *Goals*: To identify community interventions for adults that have been exposed to childhood maltreatment and have not received any sort of interventions. *Method*: First phase of Delphi method will be applied. Qualitative interviews will be conducted to at least five international recognized experts in the field of childhood maltreatment. The interview will cover issues such as target groups, assessment, retraumatization risk, resilience and intervention, trying to figure out how community interventions should be tailored. *Discussion*: The main goal of this exploratory study is to get expert information on how to foster resilience in adults that were exposed to childhood maltreatment and recognize themselves as impaired in any way because of those negative early experiences. We aim to present relevant expert information on how community interventions should be designed and which specific groups should be involved. Programs on social support development and emotional regulation training, delivered at primary healthcare systems, education institutions and media may be regarded as possible interventions. Further data will be collected in a larger group of professionals (50) following a quantitative questionnaire to better define the most suitable methods and techniques that may be applied in different cultural settings.

### Post-traumatic stress reactions (PTSR) in survivors from a terror attack in Norway: exploring the meaning of gender, age, traumatic exposure, and social support in an unselected and highly exposed group 9:00–9:15

#### G. Dyb^1,2^, T. Jensen^1,2^, E. Nygaard^2^, O. Ekeberg^2,3^, T. Diseth^2,3^ and S. Thoresen^1^

^1^Norwegian Centre for Violence and Traumatic Stress Studies, Oslo, Norway; ^2^University of Oslo, Oslo, Norway; ^3^Oslo University Hospital, Oslo, Norway

*Background*: Some characteristics of the shooting at Utøya Island in Norway July 22, 2011 allow for investigation of risk and protective factors less influenced by confounding than many trauma studies. The event was geographically isolated, all survivors could easily be identified, exposure was potentially similar across age and gender, and probably not related to pre-existing vulnerability. *Aim*: The aims were to identify if exposure was unrelated to age and gender; to investigate associations between gender, age, and PTSR; and to investigate if social support could buffer against PTSR. *Methods*: Sixty-six percentage of survivors from the Utøya Island (*N*=325) participated in interviews 5 months after the shooting. Trauma exposure was measured by questions relating to life threat, witnessing, sensory impressions, loss of someone close, and physical injuries. Social support was measured by Duke-UNC FSSQ; and peri-traumatic reactions and current PTSR by UCLA Post-Traumatic Stress Disorder Reaction Index (PTSD-RI). Multiple linear regression models were applied. *Results*: Trauma exposure was very high, e.g., 64% witnessed someone get injured or killed, and 87% reported to have seen dead people. Being aimed at/shot at, witnessing experiences, sensory impressions, physical injuries or loss of someone close were unrelated to gender and age. Female gender and young age was associated with higher PTSR, as were physical injuries and loss of someone close, while social support was associated with less PTSR. *Conclusions*: Our results strongly support vulnerability for PTSR in women. Physical injuries and loss of someone close reflected additional strain in following the shooting. The results also indicate that social support is an important buffer even following extreme stressful situations.

### Long-term mental health needs after the mass violence of 9-11-01 9:15–9:30

#### A. Naturale ICF International, Fairfax, VA, USA

The 10th anniversary of September 11, 2001 created a high level of anticipatory anxiety for victims’ families, survivors, and responders who live with the effects of this mass disaster every day. Opening the 9-11 Memorial in NYC was an additional trigger for many still coping up with the aftermath of the attacks including the war with Iraq costing thousands more lives and a stark change in key policies, changing the perception of the United States across the globe. Little empirical evidence informs long-term disaster behavioral health needs especially for human-made disasters which are known to have different and more marked consequences than natural disasters (Norris et al., 2002). Disaster research is difficult and the effort to follow the 9-11 population over time has proved overwhelming. The work of the World Trade Center Health Registry tells us that “psychological distress and psychopathology in WTC workers greatly exceed population norms and that surveillance and treatment programs continue to be needed” for those who responded to the attacks in NYC (Charney et al., 2008). To elicit the mental health needs of victims’ families, survivors, and responders during the 9-11 tenth anniversary, the Healing and Remembrance program conducted a Needs Assessment and Follow Up Survey. This paper will share the findings that informed the identification and delivery of mental health services at each disaster site.

References

Norris, F. H., Friedman, M. J., Watson, P. J., et al. (2002). 60,000 disaster victims speak: part I. An empirical review of the empirical literature, 1981–2001. *Psychiatry*, *65*, 207–239.

Charney, D. S., Herbert, R., Moline, J., Luft, B. J., Markowitz, S., Udasin, I., et al. (2008). Enduring mental health morbidity and social function impairment in world trade center rescue, recovery, and cleanup workers: The psychological dimension of an environmental health disaster. *Environmental Health Perspectives*, *116*(9), 1248–1253.

### Childhood maltreatment and the risk for revictimization and PTSD in Portuguese community subjects 9:30–9:45

#### A. Dias^1,2^ and R. Kleber^3,4^

^
1^Faculty of Social Sciences, Utrecht University, Utrecht, The Netherlands; ^2^Centro de Trauma, CES, Coimbra, Portugal; ^3^Faculty of Social Sciences, Utrecht University, Utrecht, The Netherlands; ^4^ArQ Research Foundation, The Netherlands

*Background*: Childhood maltreatment (CM) is a recognized problem in developed and developing countries with associated health consequences and large economical costs. It has been found to be connected with mental health disorders, particularly posttraumatic stress disorder (PTSD) through direct effects or through an increased revictimization probability. *Objective*: Our study aims to analyze the effects of CM exposure on the risk for revictimization and for PTSD diagnosis in community subjects in Portugal. *Method*: Cross-sectional data on PTSD and CM exposure were collected in 1200 Portuguese community subjects. The *Post Traumatic Diagnostic Scale* was used to assess the exposure to adverse events and the PTSD symptoms. The *Childhood Trauma Questionnaire Short Form* was used to assess the self-reported exposure to CM. Statistical procedures included odd ratios analyses and regression analyses. *Results*: CM-exposed subjects had an increased risk for revictimization [odd ratio=2.626 CI 95% (1.771, 3.892)] and an increased risk for PTSD diagnosis [odd ratio=2.765% (1.106, 6.910)]. Thirteen percentage of the PTSD severity was predicted by the CM exposure, namely emotional abuse (*B*=0.248, *p*=0.000) and physical neglect (*B*=0.251, *p*=0.000). Emotional neglect predicted negatively the PTSD symptom severity (*B*=−0.109, *p*=0.015). *Discussion*: Our findings confirm the association between CM, revictimization and PTSD diagnosis. CM exposure increased the risk for revictimization and for PTSD. Emotional abuse and physical neglect are significant predictors of PTSD symptoms severity. We discuss the various implications of our findings as well as the limitations and strengths of this type of research. Specific interventions should be tailored not only for specific populations at risk, but also for community subjects exposed to CM, addressing the revictimization and the PTSD symptoms as well.

### Adult attachment strategies as predictors of long-term symptomatic outcome following child abuse experiences: comparing and contrasting patterns following child physical, sexual and/or emotional abuse 9:45–10:00

#### P. Petretic, M. Karlsson, M. Calvert and J. Henrie Psychology Department, University of Arkansas, Arizona, USA

Studies of post-abuse outcome in adults who have experienced child abuse have documented variability of long-term distress. Subsequently, the impact of moderating variables that may impact outcome, including the construct of attachment, has been investigated. The current study evaluated the predictive value of 11 attachment strategies/styles on current symptoms of distress in young adults reporting a history of single (physical, emotional, sexual) or multiple forms of child abuse and those without this history. Respondents (*N*=762) completed measures assessing abuse history, attachment styles, and strategies (Experiences in Close Relationships—extended research version; ECR-R), and trauma symptoms (Trauma Symptom Inventory; TSI) MANOVAs indicated significant differences between abuse groups and the no-abuse control group for interpersonal attachment styles (both positive and negative strategies) as well as symptomatic distress. Adults reporting multiple forms of abuse (physical and emotional), sexual abuse, and emotional abuse demonstrated more severe adult attachment impairment in several areas, specifically: being unable to have a sense of trust in others, not viewing oneself as good and worthy of love, and having a style that is uncomfortable with emotional intimacy, being affectionate and close to a partner, with the first two groups also demonstrating significantly greater symptomatic severity. Multiple regressions indicated that specific attachment strategies predicted a significant amount of variance across all three symptomatic domains (trauma, self, and dysphoria) for these three abuse groups. While lovability predicted all symptom clusters for sexual abuse victims, uncertainty, and trust predicted symptom clusters for physical and multiple abuse groups. Unique predictors for dysphoric distress varied from those for trauma and self-distress clusters. Findings suggest that assessing and potentially targeting interpersonal functioning/attachment, specifically perceptions of other and self in current primary intimate relationships, may be of considerable value in treatment of adults presenting with a history of personal distress following childhood abuse.

## Open Papers: Cultural issues

### Traumatic postmemory: the children of the Colonial War 10:15–10:30

#### A. Sousa Ribeiro^1^, L. Sales^2^, M. Calafate Ribeiro^3,4^ and A. Dias^5,6^

^1^School of Arts and Humanities and Centre for Social Studies, University of Coimbra, Coimbra, Portugal; ^2^Coimbra Military Hospital and Centre for Social Studies, University of Coimbra, Coimbra, Portugal; ^3^Centre for Social Studies, University of Coimbra, Coimbra, Portugal; ^4^Centre for Social Studies, University of Bologna, Bologna, Italy; ^5^Faculty of Social Sciences, Utrecht University, Utrecht, The Netherlands; ^6^Centro de Trauma, CES, Coimbra, Portugal

Drawing on the results of a transdisciplinary research project aimed at analysing the transgenerational effects of trauma among children of former participants in the Portuguese Colonial War (1961–1974), the paper will pursue two lines of inquiry: first, the perspective of psychology, an analysis of the responses to several psychometric questionnaires (*Impact Event Scale Revised*, *Posttraumatic Diagnostic Scale*, *Childhood Trauma Questionnaire, Young Schema Questionnaire*, *Brief Symptom Inventory*) which provide clear evidence of the increased vulnerability to trauma of the second generation; second, a discourse-analytic approach to a wealth of collected interviews, on the one hand, and, on the other hand, to other types of testimony as expressed, in particular, in the discourse of literature and the arts. The results of such a combined approach provides fresh insights to show how the overwhelming public silence on the most traumatic experienced in recent Portuguese history may be countervened by the emergence of a multitude of voices which are awaiting to be heard and which reject the position of the victim by claiming a status of authorship.

### Resettlement and mental health of Bosnian refugees in Austria and Australia: the impact of acculturation 10:30–10:45

#### D. Kartal^1^ and L. Kiropoulos^2^

^1^Monash University, Melbourne, Australia; ^2^University of Melbourne, Melbourne, Australia

Traumatic stress brought by traumas such as war and displacement are found to have a long lasting impact on the psychological well-being of individuals, with Posttraumatic Stress Disorder (PTSD) and depression particularly prevalent in refugee populations. Despite the high prevalence rates of mental health disorders research have infrequently investigated the role of acculturation stress. The current study examines the relationship between traumas, acculturation factors, and mental health symptoms in Bosnian refugees resettled in Austria and Australia. It was postulated that apart from trauma exposure, different resettlement stressors, and affiliations such as acquisition of language, discrimination, acculturation strategy, and identification with one's own culture could influence or even mediate the relationship between trauma and mental health. Using snow-balling strategy, approximately 70 data sets were obtained. Prevalence of depression, anxiety, and PTSD symptoms were measured using the PSS-SR and DASS. Acculturative strategies, attitudes, and behaviours toward the host and national cultures were measured with multiple scales (DIS, LIB). Additional questions were included to explore refugees’ experiences of war, displacement, and the continuing stressors of resettlement. Hierarchical regressions were used to investigate the relationship between trauma, acculturation, and mental health outcomes. The preliminary findings will be discussed. These findings are expected to assist community agencies, policy makers and mental health workers working with refugees and asylum seekers, by providing them with effective and appropriate support on cultural, individual, and community levels.

### Literature as a way to overcome past traumas: some examples from Cyprus and Germany 10:45–11:00

#### H. Karahasan Girne American University, Kyrenia, Cyprus

Although trauma is a personal thing, it can be collective too. Using some literary works as examples, this paper will show how literature can be used to overcome past traumas. Uwe Timm's *In the Shadow of My Brother* is a good example of how a literary work can be used to overcome the past trauma of the Nazi Germany as well as Timm's personal life. In that work, Timm departs from his relationship between his older brother, who fought for the Nazis, and explore the issue as how both his own brother as well as the entire country fall to Nazi ideology, which is a trauma for th German community. The case of Cyprus is a bit different than Timm's Germany. In Cyprus, recent interethnic violence led the two main communities in Cyprus to live separately since 1974. This paper will focus on some literary works of the “74 Generation” of Cypriot writers and show how these works can be seen as examples of overcoming the trauma of war in Cyprus. Mehmet YaşIn, Nese YaşIn, and Faize Özdemirciler are just few poets who belonged to this generation with their works. In brief, the paper will show how literature can be used to overcome past traumas with the examples of Cypriot writers as well as Uwe Timm's works.

### Posttraumatic survival—the lessons of Cambodian resilience 11:00–11:15

#### G. Overland Regional trauma centre (RVTS) of Southern Norway, Kristiansand, Norway

What lessons do successful posttrauma survivors have to teach us? This paper examines the field of cultural knowledge through the eyes and with the vocabulary of interviewees: adult Cambodian survivors of the Khmer rouge régime who have managed remarkably well. In the process of multiple back-translation of the interview data it became clear that a number of central terms could in some way be bearers of answers to the salutogenic question: why they had managed so well. This suggested the need for a more intensive analysis of these unfamiliar terms—an exegetical approach like that used by Mollica in the study of trauma narratives (2006). The paper will give empirical examples from the data, the words and expressions in question, and follow the argumentation leading to the conclusions: the centrality for informants of cultural responses, embedded in their everyday vocabularies, to traumatic events in their pasts.

